# Wings of Change: aPKC/FoxP-dependent plasticity in steering motor neurons underlies operant self-learning in *Drosophila*

**DOI:** 10.12688/f1000research.146347.1

**Published:** 2024-02-19

**Authors:** Andreas Ehweiner, Carsten Duch, Björn Brembs

**Affiliations:** 1 Institut für Zoologie - Neurogenetik, Universität Regensburg, Regensburg, Bavaria, 93040, Germany; 2 Institute of Developmental Biology and Neurobiology (iDN), Johannes Gutenberg Universitat Mainz, Mainz, Rhineland-Palatinate, Germany

**Keywords:** insect, flight, learning, memory, operant, molecular, gene, CRISPR

## Abstract

**Background:**

Motor learning is central to human existence, such as learning to speak or walk, sports moves, or rehabilitation after injury. Evidence suggests that all forms of motor learning share an evolutionarily conserved molecular plasticity pathway. Here, we present novel insights into the neural processes underlying operant self-learning, a form of motor learning in the fruit fly *Drosophila.*

**Methods:**

We operantly trained wild type and transgenic *Drosophila* fruit flies, tethered at the torque meter, in a motor learning task that required them to initiate and maintain turning maneuvers around their vertical body axis (yaw torque). We combined this behavioral experiment with transgenic peptide expression, CRISPR/Cas9-mediated, spatio-temporally controlled gene knock-out and confocal microscopy.

**Results:**

We find that expression of atypical protein kinase C (aPKC) in direct wing steering motoneurons co-expressing the transcription factor *FoxP* is necessary for this type of motor learning and that aPKC likely acts via non-canonical pathways. We also found that it takes more than a week for CRISPR/Cas9-mediated knockout of *FoxP* in adult animals to impair motor learning, suggesting that adult *FoxP* expression is required for operant self-learning.

**Conclusions:**

Our experiments suggest that, for operant self-learning, a type of motor learning in *Drosophila*, co-expression of atypical protein kinase C (aPKC) and the transcription factor *FoxP* is necessary in direct wing steering motoneurons. Some of these neurons control the wing beat amplitude when generating optomotor responses, and we have discovered modulation of optomotor behavior after operant self-learning. We also discovered that aPKC likely acts via non-canonical pathways and that FoxP expression is also required in adult flies.

## Introduction

Motor learning is an essential component of human behavior and is ubiquitous throughout the animal kingdom. The process of learning a motor skill can be influenced by a number of factors, such as the amount of training, type of feedback, or the presence/absence of environmental cues. Regaining lost motor functions after brain or spinal cord injury is considered a crucial component of rehabilitation. Human language is acquired by a form of motor learning, and motor learning also appears to be a key invention that allowed the newly evolved animals in the Cambrian to become ambulatory. ^1^^–^^3^ Vocal learning, such as learning to speak, is a form of motor learning ^4^ that involves the Forkhead Box transcription factor family P (FoxP) in vertebrates. ^5^^–^^15^ Other, nonvocal forms of motor learning also involve FoxP genes in vertebrates ^16^^–^^19^ and invertebrates. ^20^ Vocal learning also shares the involvement of protein kinase C (PKC) with other forms of motor learning, ^21^^–^^25^ raising the possibility of a conserved motor learning pathway extending beyond these two components.

While motor learning shares various features with other forms of learning, such as operant learning producing habits or skill-learning, it is debated how many common biological mechanisms these different concepts share. ^26^^–^^29^ Understanding motor learning in a numerically smaller nervous system in a genetically tractable organism where one can not only study the motor learning process itself, but also its interactions with other forms of learning, ^30^ may help inform these debates.

Here we provide further evidence about the specific manner in which FoxP and PKC are involved in a form of motor learning in the fruit fly *Drosophila*, operant self-learning at the torque meter. In this experiment, motor learning dissociates from other forms of learning such that genes involved in motor learning are not involved in other forms of learning and *vice versa.* ^31^^–^^33^ At the torque meter, a fly is tethered between head and thorax such that it can move all other appendages. When beating its wings, the fly generates forces, some of which can be measured by the torque meter. Specifically, the torque meter measures torque around the vertical body axis, yaw torque. ^34^ Even in the absence of any guiding cues, flies can learn to associate one torque domain (e.g., roughly corresponding to left or right, respectively, turning maneuvers) with a punishing heat beam. ^35^ This experiment not only conceptually mimics other motor learning paradigms in that feedback is made immediately contingent on specific motor actions, but also via its dependence on *FoxP* and PKC genes. ^20^^,^^21^ This form of motor learning has been termed operant self-learning to distinguish it from other forms of operant learning and to denote that the subject is learning about its own behavior, as opposed to some stimulus associated with the behavior. ^32^

For operant self-learning in *Drosophila*, it is not known in which neurons the *FoxP* gene is required and which PKC gene is involved. It is also unknown which pathway is engaged by PKC and whether FoxP expression is also required acutely in adult flies for this form of motor learning. In this work, we addressed all three research questions.

## Methods

### Strains and fly rearing

If not stated otherwise, flies were raised on standard cornmeal/molasses medium ^36^ at 25°C and 60% humidity under a 12-hour light/dark cycle. For experiments requiring the expression of temperature-sensitive Gal80, animals were raised at 18°C. To set up crosses for behavioral experiments, 20 females were placed together with five to eight males and were allowed to lay eggs for 24 h. They were flipped daily into fresh vials, to ensure appropriate larval density. In 30 years of research on learning and memory in tasks like ours, no difference was ever observed between male and female flies in terms of learning ability. Whenever genetically appropriate, we used female flies for practical reasons. The flies were prepared the day before the experiment, allowing them time to recover ( Table 1). Female flies (24 to 48 h old) were briefly immobilized under cold anesthesia. A thin triangular copper hook (0.05 mm diameter) was glued (3m Espe Sinfony, 3M Deutschland GmbH) between head and thorax, fixing both body parts to each other. ^37^ Each animal was kept individually in a small moist chamber with a few grains of sugar. For *tub-Gal80 ^ts^* expression, animals were raised at 18°C and incubated at 30°C for two days. Experiments were always conducted at room temperature. For experiments using the gene-switch system, newly hatched flies were placed on *Drosophila* instant medium (351.204, Schlüter Biologie, Eutin-Neudorf, Germany) containing the steroid hormone RU486 (200 μg/ml, CAS No.: 84371-65-3, Sigma-Aldrich, St. Louis, MO) for two days.

**Table 1.** Fly strains used in this work.

Genotype	use	Bloomington	Flybase
;;nSyb-GS	driver	80699	FBti0201287
;;GMR55G08-GAL4	driver	50422	FBti0136906
;;GMR65A06-GAL4	driver	39330	FBti0137511
;;GMR20H05-GAL4	driver	47896	FBti0133817
;;ato-Gal4	driver		
ELAV-Gal4;;	driver		
ELAV-Gal4;Tub-Gal80ts;;	driver		
;;FoxP-iB-Gal4/TM3	driver		
;;FoxP-LexA	driver		
;;UAS-g-aPKC	effector	85862	FBti0210993
;;UAS-g-BAZ	effector	84234	FBti0207133
;;UAS-g-PKC53e	effector	76612	FBti0194968
;UAS-g-KIBRA	effector		
;;UAS-Cas9;	effector		
;;UAS-PKCi	effector	4589	FBti0010565
;;UAS-t:gRNA(4xFoxP)	effector		
;UAS-Cas9;;	effector		
C380-Gal4;;	driver	80580	FBti0016294
y[1] w[*]; Mi {Trojan GAL4.un} aPKC [MI10848-TG4.un]/SM6a	driver	77814	FBti0196316
LexAop-mD8-RFP-UAS-mCD8-GFP;TM3/TM6b	effector		
Wild type Berlin	wild type strain		

The UAS-g-KIBRA line was generated in the laboratory of Michael Krahn, Universität Münster ( Table 1). They cloned the following guide RNAs from KIBRA Intron-2: GTACTTACGACTGCTTCGAC and KIBRA Intron-4: GGGCACCGTGCAGATCAGCA in pCDF6 and inserted them in attP40.

### Behavioral setups

Two different torque meters and setups had to be used for technical reasons, not by choice. Both torque meters were described previously: The ‘Tang’ meter ^38^ and the ‘Götz’ meter ^34^(RRID:SCR_017276), named after the authors. With flies attached to the torque meter via a clamp, all devices measure the rotational force (torque) around the animal’s vertical body axis. While the Götz meter is older than the Tang meter, it is technically more advanced because of its rotational compensation, and was included in a more modern version of the setup that was used in the later experiments, after the first data were collected using the Tang setup. Even later, the wild type experiments shown in Figure 4 were conducted with a third torque meter, still in the prototype phase, which combines the laser-based measurement of the Tang meter with induction-based compensation of the Götz meter. Documentation of this setup is in preparation, but the operating principles remain the same as for the two referenced devices. In all setups, the animal is surrounded by a cylindrical panorama (arena; diameter 58 mm Tang, 90 mm other setups), homogeneously illuminated from behind by either a projector (new setups: DLPLCR4500EVM, Texas Instruments) or a halogen lamp (Tang: OSRAM 100W/12V), such that stationary flight in a controlled environment was achieved. An infrared laser (Stocker Yale Lasiris SNF series; 825 nm, 150 mW) was used as punishment in all setups. The laser was pointed from above onto the animal’s head, pulsed (approximately 200 ms pulse width ~4 Hz) and the intensity adjusted empirically for maximal heat avoidance and learning. The experiment is fully computer controlled, using custom software (Tang: LabView, National Instruments, RRID:SCR_014325. New setups: DOI: 10.5281/zenodo.7102195).

### Design of behavioral experiments

Before each self-learning experiment, the yaw torque range was adjusted using optomotor stimuli for each fly tethered to the torque meter. The optomotor response (OMR) is an innate, orienting behavior evoked by whole-field visual motion and is common to vertebrates and invertebrates. The OMR has algorithmic properties such that the direction of the whole-field coherent motion dictates the direction of the behavioral output (e.g., leftward visual stimuli lead to turning left, and rightward visual stimuli lead to turning right). For instance, when tethered Drosophila are surrounded by vertical black and white grating patterns rotating along the fly’s azimuth (i.e, around the fly’s vertical body axis), the fly will turn (i.e., produce yaw torque) in the direction of perceived motion. Typical OMRs for tethered flies responding to horizontally rotating vertical stripes are depicted in Figure 4A1.

For the ‘Tang’ setup, arena rotation for the optomotor stimulus was operated by hand. The direction of the rotation was reversed after the fly reached its asymptotic optomotor torque. During optomotor presentations before the self-learning experiment, the torque was adjusted to be zero-symmetric. This was to facilitate unbiased torque preferences before training. Torque traces during OM presentations were not stored in experiments using the ‘Tang’ setup. Optomotor stimuli (15 vertical black stripes on a white background taking about 3.5s for a full rotation, i.e., a pattern wavelength of 24° at a pattern frequency of about 4.3 Hz) were presented for a duration of 30 s in each turning direction for flies in the new setups and recorded in the raw data files together with all other data from each experiment. Because of this difference, as experiments recorded with the Tang device did not record optomotor periods, periods in ‘Tang’ experiments are numbered from 1 to 9 ( Table 2), while optomotor periods for the new setups were included, such that the periods in the new setups were numbered 1-17 ( Table 3). Because we used four optomotor periods each before and after training in the new setup, periods 5 to 13 were the periods in which self-learning was studied in this setup.

**Table 2.** Experimental sequence “Tang Setup”. All periods lasted 120s.

Period 1	Period 2	Period 3	Period 4	Period 5	Period 6	Period 7	Period 8	Period 9
Pretest	Pretest	Training	Training	Test	Training	Training	Test	Test
No heat	No heat	**Heat**	**Heat**	No heat	**Heat**	**Heat**	No heat	No heat

**Table 3.** Experimental sequence for new setups. OM - optomotor. Torque learning periods (5-13) lasted 120s, while OM periods lasted 30s.

OM before				Experiment									OM after			
Period 1	Period 2	Period 3	Period 4	Period 5	Period 6	Period 7	Period 8	Period 9	Period 10	Period 11	Period 12	Period 13	Period 14	Period 15	Period 16	Period 17
OM	OM	OM	OM	Pretest	Pretest	Training	Training	Test	Training	Training	Test	Test	OM	OM	OM	OM
No heat	No heat	No heat	No heat	No heat	No heat	**Heat**	**Heat**	No heat	**Heat**	**Heat**	No heat	No heat	No heat	No heat	No heat	No heat

The main self-learning experiment then consisted of nine periods of two minutes duration in both setups. The laser was permanently off during the first two periods, so that the fly could freely choose its direction of turning maneuvers without any feedback. In the following two training periods either the left or the right torque domain was associated with the punishing laser, without any hysteresis. The punished torque domain was alternated between experiments. The first two training periods were followed by one test period without punishment. Afterwards, the fly was trained again with the same side punished as before for another two 2-min. periods. Finally, no heat was applied in the final two test periods, allowing the fly to express its spontaneous yaw torque preference. The figures always show the preference in the first test period after the last training period, i.e., period 8 (performance index, PI8) in the Tang setup and period 12 (PI12) in the new setups ( Tables 2, 3). When the axis labels in the figures differ with regard to PI8 or PI12, these differences only indicate which setup was used. In all cases, the same first test period after the last training period was used to test for learning, irrespective of setup.

### Data handling and statistical analysis of behavioral experiments

***Data selection***

To ensure proper punishment by the laser, each fly was exposed to the laser after the experiment, to ensure it was adjusted correctly. If the fly survived the laser for 15 s or longer, the data were excluded from analysis. Data that did not show any or shifted OMRs, indicating either an unhealthy fly or an error with the measuring device, were also excluded. Data were also excluded if the fly had not experienced the laser at least once during training. Finally, flies with poor flight performance (constant stopping of flight) were also excluded from analysis. While these data were excluded from analysis, all complete traces are nevertheless included in the published data sets, such that the inclusion criteria can be independently tested.

***Data availability and analysis***

The preference of a fly for right or left torque domain was quantified as the performance index PI = ( *ta* − *tb*)/( *ta* + *tb*). During training periods, *tb* indicates the time the fly is exposed to the heat and *ta* the time without heat. During tests, *ta* and *tb* refer to the times when the fly chose the formerly (or subsequently) unpunished or punished situation, respectively. Thus, a PI of 1 indicates the fly spent the entire period in the situation not associated with heat, whereas a PI of −1 indicates that the fly spent the entire period in the situation associated with heat. Accordingly, a PI of zero indicates that the fly distributed the time evenly between heated and non-heated situations and a PI of 0.5 indicates that 90 of the 120 s in that period were spent in the unpunished situation.

Analogously, optomotor ( Table 3, OM) behavior was quantified by computing an OM asymmetry index from the torque traces. Straight lines or double sigmoidal models were fitted to individual torque traces from OM periods, depending on the detected slope of the OMR. Each fit was generated separately for each turning direction. From the fitted lines/models, optomotor magnitude was derived as either the intercept (lines) or the asymptote (double sigmoidal model) for each turning direction. The magnitude of left-turning torque was subtracted from the magnitude of right-turning torque and divided by the sum of the two values. This optomotor asymmetry index becomes -1 for OMRs where clockwise (‘right-turning’) stimuli elicit no or left-turning torque (while counter-clockwise stimuli elicit left-turning torque). It becomes 1 for OMRs where counter-clockwise (‘left-turning’) stimuli elicit no or right-turning torque (while clockwise stimuli elicit right-turning torque). The OM asymmetry index becomes zero if the absolute magnitudes of OMRs in both directions are equal. In brief, a positive optomotor asymmetry index indicates shifts away from symmetrical torque towards right-turning torque and a negative index indicates shifts towards left-turning torque.

All behavioral data were analyzed using R (R Project for Statistical Computing) (RRID:SCR_001905). The collection of R-scripts evaluating the time series data can be found at DOI 10.5281/zenodo.10041052. ^39^ The data model pertaining to the XML raw data files and the YAML data set files can be found at 10.5281/zenodo.7101734. ^39^ In brief, the XML data files contain both the meta-data for each single fly experiment as well as the time-series data covering the entire experiment. The single YAML file per dataset contains the experimental design, such as which data files belong to which experimental group, the type of statistics to be performed, significance levels used, experimenter comments and data inclusion/exclusion. The main R-Script reads the YAML dataset files and performs the appropriate computations for quality control, analysis and statistics.

Quality control is performed on each single-fly XML file and included in the published datasets. Each single-fly experiment XML file is thus accompanied by a single-fly HTML quality control report sheet containing plots of the raw time series data, as well as a number of evaluations necessary to assess the proper execution of the experiment and the quality of the resulting data.

Data analysis and statistics for each dataset are reported in an HTML dataset evaluation sheet. Thus, a complete dataset consists of one XML raw data file and one HTML quality control sheet for each fly, plus a single YAML dataset file and one HTML dataset evaluation sheet.

The datasets were published using a custom Python script (DOI: 10.5281/zenodo.7101741) ^39^ that synchronizes the collected data on the local computer with the University of Regensburg publication server. The persistent identifiers for each dataset are listed in the figure legends.

***Statistics***

Motivated by ongoing efforts to improve statistical inference in science (e.g.,, ^40^^–^^54^ we chose to statistically evaluate PIs in two complementary ways, using both frequentist and Bayesian statistics. ^55^^–^^61^ Following previous studies, ^30^^,^^31^^,^^62^^–^^65^ individual PIs of the first test after the last training period were tested against zero to evaluate the ability of the manipulated flies to show a preference towards the unpunished torque domain. The rationale behind estimating a group of flies as either showing learning or not is to trade-off statistical power with a more nuanced measure of learning performance: comparing between experimental groups may yield more nuance, but also requires impractically large sample sizes for adequate (>80%) statistical power. To further reduce the chance of statistical error, we used both Wilcoxon tests in a frequentist scenario and computed the equivalent Bayes Factors for a Bayesian version. We set the alpha value for the Wilcoxon test to 0.5% as suggested by, ^46^ such that p-values below 0.005 and Bayes factors above 5 for the same group of flies would be considered compelling evidence that the flies were able to learn. Conversely, Bayes Factors below one together with p-values higher than 0.05 were considered evidence that the flies were not able to show self-learning. Finally, groups where the two statistics were in conflict or intermediate, were considered inconclusive. Thus, both the Bayesian and the frequentist criteria had to be met in order to claim that a genetic manipulation interfered with operant self-learning. These criteria were chosen to quantitatively distinguish between effective and ineffective manipulations without neglecting the uncertainty associated with all experimentation. At the same time, our evaluations were chosen to specifically identify large contributions to the learning processes that can be identified with sufficient statistical power (i.e., large effect sizes). All statistical results are published with the raw data and the code used to compute them is openly available.

### Gene expression analysis in flight steering motor neuron terminals

***Dissection***

The axon terminals on flight steering muscles were tested immunocytochemically for the expression of *FoxP* and aPKC in flies expressing LexAop RFP under the control of FoxP-Lex and UAS td-GFP under the control of aPKC-trojan GAL4 (LexAop RFP, UAS td-GFP/aPKC-Trojan-GAL4; FoxP-LexA/+). In addition, synapses at neuromuscular junctions were labeled with the active zone marker *bruchpilot* ( *brp*, ^66^). Animals were dissected in normal saline along the dorsal midline and bent open with minute pins inserted through the dorsalmost edge of the dorsal longitudinal flight muscles (DLMs). The heart, gut, fat tissue, and other connective tissue were removed to expose the ventral nerve cord (VNC) and the musculature. Next, on both sides the DLMs and dorsoventral flight muscles (DVMs) were carefully removed layer-by-layer to expose the direct flight steering muscles, which are located close to the lateral cuticle of the thorax. Preparations were rinsed 5-10 times in saline to remove debris from the dissection procedure, and specimens were fixed for 1 h in 4 % paraformaldehyde in 0.1 M PBS buffer at room temperature (22°C). After fixation specimen paraformaldehyde was exchanged with 0.1 M PBS buffer.

***Immunohistochemistry of motor terminals***

Following fixation, specimens were washed 6 × 20 min in 0.1 M PBS buffer at room temperature. Next, preparations were washed 3 × 1 h in 0.1 M PBS-Tx (0.3%) buffer at room temperature and incubated with primary antibodies (mouse α-brp, 1:500 (Hybridoma bank, NC82)); chicken α-GFP (1:1000, Life Technologies A10262), and rabbit α-mCherry, (1:500, PAS-34974)) in 0.1 M PBS-Tx (0.3 %) buffer at 4° C for 24 to 36 h. Following primary antibody incubation, preparations were washed 6 × 1 h in 0.1 M PBS buffer at room temperature. Next animals were incubated in secondary antibodies: donkey α-mouse Alexa 647 (JacksonImmunoResearch 715-605-150), donkey α-chicken Alexa 488 (Dianova 703-545-155), and donkey α-rabbit Alexa 568 (Invitrogen A10042) in 0.1 M PBS-Tx (0.15%) buffer at 4°C for 24 to 36 h. Following secondary antibody incubation, preparations were washed 3 × 1 h in 0.1 M PBS buffer at room temperature, dehydrated in an ascending ethanol series (50, 70, 90, and 2 × 100 % EtOH, 15 min each), and cleared for 5 min in methyl salicylate. Finally, preparations were mounted in methyl salicylate in between two coverslips that were glued onto both sides of a round hole (10 mm diameter) drilled into custom-made metal slides of 188 μm thickness. ^67^ Briefly, one cover slip was fixated with superglue underneath the hole, the space was filled with methyl salicylate, the preparation transferred into the mounting media, and another coverslip was carefully placed on top of the hole, so that no air remained in the methyl salicylate filled hole. The top coverslip was fixed to the metal slide using transparent nail polish (DM Markt, Mainz, Germany). After 20 min to let the nail polish dry the preparation was transferred to the microscope. In total 11 animals were subjected to immunocytochemistry on direct flight muscles. In nine of these 11 preparations, we were able to identify only subsets of the direct wing steering muscles under investigation. These subsets yielded identical results with respect to aPKC and FoxP expression as the two preparations with complete sets of direct muscles as shown in Figure 3.

***Confocal laser scanning microscopy***

Preparations were scanned using a Leica (Leica Microsystems, Germany) SP8 confocal laser scanning microscope (CLSM) under either a 20x oil (NA = 0.75) or a 40x oil (NA = 1.3) lens at an image format of 1024 × 1024 pixels. Z-step size was 2 μm for the 20x lens (resulting in voxel dimensions of 0.57 × 0.57 × 2 μm) and 1 μm for the 40x lens (resulting in voxel dimensions of 0.28 × 0.28 × 1 μm). Alexa 488 was excited with an argon laser at 488 nm and detected with a photomultiplier between 495 and 530 nm wavelength. Alexa 488 was excited with a solid state laser at 561 nm and detected with a photomultiplier between 570 and 610 nm wavelength. Alexa 647 was excited with a red helium neon laser at 633 nm and detected with a photomultiplier between 640 and 670 nm wavelength. All image stacks were stored as.lei files and further analyzed using Las X software (Leica Microsystems, Germany). Selected fields of view were used for maximum intensity projection views, which were exported as 24 bit three color tiff images and further processed with Corel Draw 11.

***Data availability***

Confocal image stacks can be found at: 10.5281/zenodo.10606166. ^68^

## Results

### Self-learning requires a *PKC* in *FoxP* motor neurons

**Figure 1.**
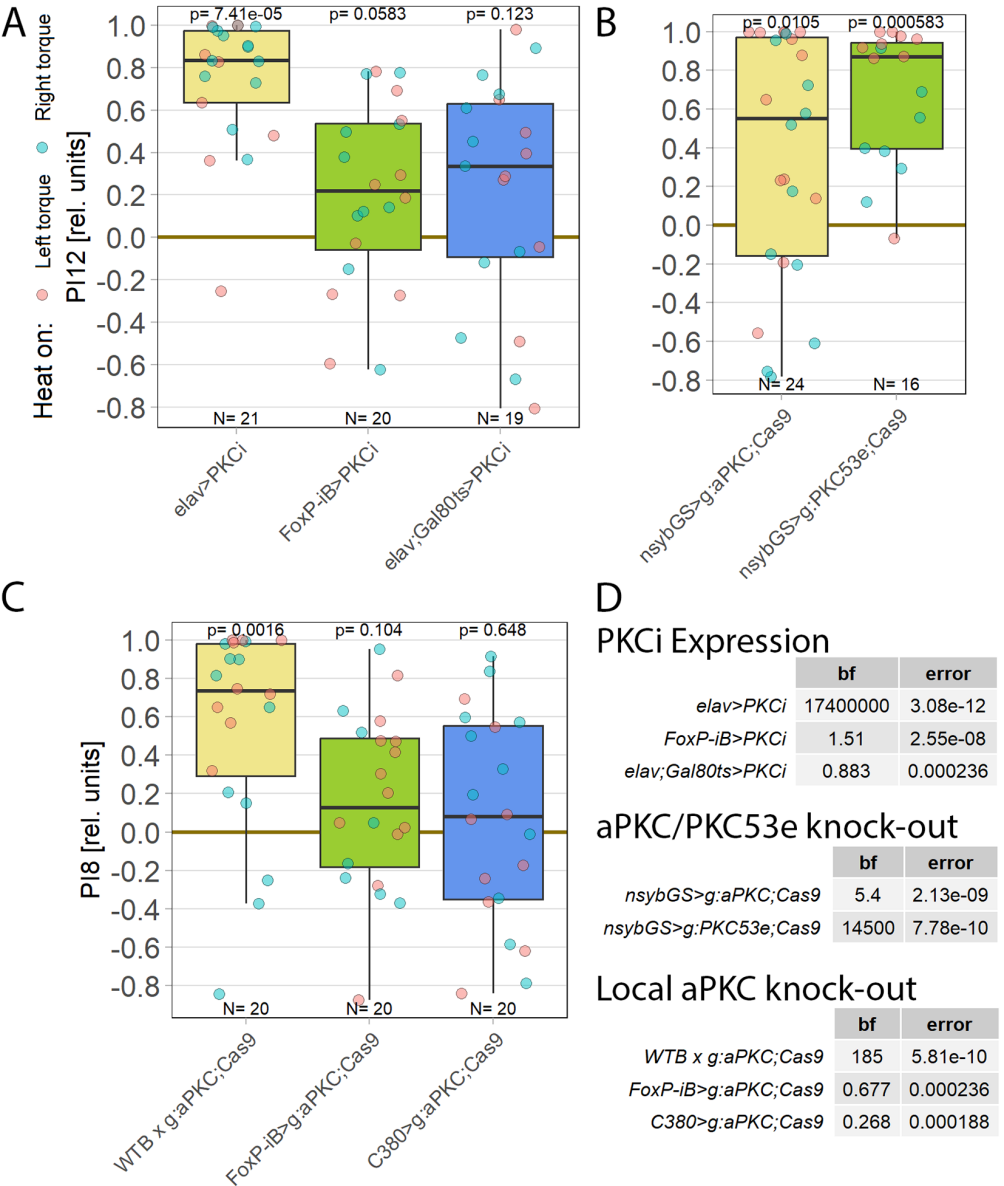
For operant self-learning, *aPKC* is required in motor neurons. Performance indices for the first period after training (PI8/PI12) are plotted. Each colored dot represents a single fly experiment. Red dots denote experiments where the fly was punished on its left turning torque domain, blue dots denote flies that were punished on their right turning domain. Box plots denote medians, quartiles and non-outlier range. Statistical analyses test for differences of PIs against zero. A. Inhibiting all protein kinase C isoforms with the inhibitory peptide PKCi. Constitutive, pan-neuronal expression PKCi (left, yellow), leads to high PIs and a large Bayes Factor, indicating this manipulation left self-learning intact. Expressing PKCi either in FoxP-isoform B positive neurons without temporal control (middle, green), or in all neurons but restricted to adulthood using *tub-Gal80 ^ts^* (right, blue, see Methods for details) yields low PIs, high p-values and low Bayes factors, indicating self-learning was impaired. Data: 10.5283/epub.52958. ^74^ B. Pan-neuronal knock-out of two different PKC genes with CRISPR/Cas9 in adulthood (using the GeneSwitch system and feeding RU486 to adult flies, see Methods for details) suggests aPKC is necessary for operant self-learning. Knocking out atypical PKC (yellow, left) yields moderate PIs, p-values and Bayes factors, indicating some effect on operant self-learning, while the high PIs, low p-values and high Bayes factor of the group where PKC53E was knocked out (right, green) indicate their self-learning was intact. Data: 10.5283/epub.52957. ^75^ C. Knocking out aPKC in motor neurons or *FoxP*-neurons impairs operant self-learning. Expressing the CRISPR/Cas9 components either in *FoxP* isoform B-positive neurons (green, middle) or in motor neurons (blue, right) leads to low PIs, high p-values and low Bayes Factors, indicating their self-learning is strongly impaired. Control flies with only the CRISPR/Cas9 genetic elements but no driver, showed high PIs, a low p-value and a high Bayes Factor, indicating their self-learning was intact. Data: 10.5283/epub.52944. ^76^ D. Bayesian statistics for the three datasets.

Colomb and Brembs (2016) discovered that blocking all protein kinase C (PKC) isoforms in neurons using the inhibitory peptide PKCi abolished operant self-learning. We replicated their results by pan-neuronal expression of PKCi. In one group, PKCi was expressed both during development and in the adult flies. In the other group, PKCi expression was restricted to adulthood only, by using the temperature-sensitive Gal4 inhibitor *tub-Gal80 ^ts^* (see Methods for details). Temporally unrestricted pan-neural expression did not impair self-learning, whereas expression restricted to neurons in adult flies abolished self-learning ( Figure 1A). This result may seem surprising, but compensation for experimental manipulations of PKC activity through development has been reported numerous times in the literature ^69^^–^^73^ and our data reproduced our previously published, identical experiments with PKCi, ^21^ demonstrating the PKCi construct is still performing as expected. Expressing PKCi only in cells expressing isoform B of the FoxP gene also abolished self-learning, even without temporal control, suggesting that PKC activity is required in FoxP-positive neurons.

Colomb and Brembs (2016) also discovered that restricting PKCi expression to motoneurons (MNs) was sufficient to impair self-learning. The FoxP-iB driver line replicated these results as well, as *FoxP* isoform B is prominently expressed in motor neurons. ^77^ To help determine which PKC may be involved in this mechanism, we screened RNA-Seq databases for PKC genes expressed in MNs. Restricting candidates to those where gRNA lines were available for CRISPR/Cas9-mediated gene knockout yielded only two genes: the atypical PKC (aPKC) and the diacylglycerol-activated PKC53E. Knocking out each gene pan-neuronally in adult flies and testing the manipulated animals for operant self-learning showed excellent learning performance in PKC53E-manipulated flies ( Figure 1B), clearly ruling out PKC53E as the gene involved in operant self-learning. The performance of the aPKC-manipulated flies was inconclusive: their preference scores were somewhat lower, not reaching our criteria for significant learning, but at the same time too high to be confident in the result ( Figure 1B).

As PKC activity is required in MNs, ^21^ we limited the aPKC knockout to these neurons which abolished self-learning ( Figure 1C). To test the hypothesis articulated above that aPKC activity is required in *FoxP* neurons, we also knocked out aPKC in *FoxP* neurons, which also abolished self-learning. ( Figure 1C). Using two different driver lines also controls for driver-specific effects and potential expression outside of motor neurons. Both driver lines support operant self-learning in principle (article in preparation, data at DOI: 10.5283/epub.52962), with C380-Gal4 also already in the peer-reviewed literature. ^21^ To our knowledge, C380 and FoxP-iB expression overlaps only in MNs.

### *aPKC* and *FoxP* are co-expressed in identified flight steering MNs

**Figure 2.**
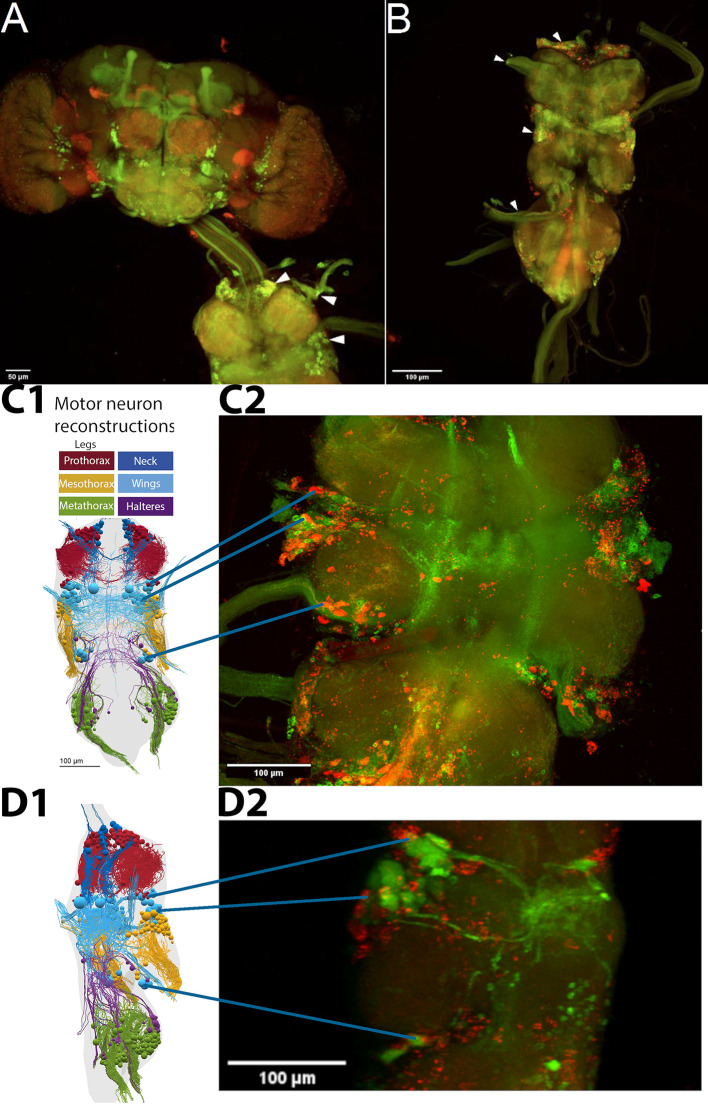
FoxP and aPKC are co-expressed in MNs. Confocal stacks of whole mount preparations of central nervous systems; A-C: green - aPKC-Gal4>CD8::GFP, red - FoxP-LexA>CD8::RFP; D: green - D42-Gal4>CD8::GFP, red - FoxP-LexA>CD8::RFP. Confocal image stacks available at: 10.5281/zenodo.10047941. A. Adult brain (top) with ventral nerve cord (VNC, bottom) attached. No co-expressing cells can be observed in the brain, whereas such neurons (yellow) are readily observable in all neuromers of the VNC (arrowheads). B. VNC with *aPKC/Foxp* co-expression (yellow) both in cell bodies and fiber tracts in nerves (arrowheads). C. C1: Dorsal view of motor neuron reconstruction (modified from Ref. 78) C2: Confocal image stack of dorsal view of the mesothoracic neuromer with putative wing MNs expressing both *aPKC* (green) and *FoxP* (red) marked. D. D1: Lateral view of motor neuron reconstruction (modified from Ref. 78). D2: Confocal image stack of mesothoracic neuromer with all MNs (green) and FoxP neurons (red) marked. This lateral view supports the hypothesis that the ventral cluster of *aPKC/FoxP* neurons comprises wing MNs.

Thus, the behavioral data presented above support the hypothesis that the plasticity mediating operant self-learning takes place in neurons that co-express both *FoxP* and *aPKC.* Whole-mount confocal microscopy of fly central nervous systems with *aPKC*-Gal4 and *Foxp*-LexA expression suggested that neurons expressing both *aPKC* and *FoxP* exist only in the ventral nerve cord (VNC, Figure 2A, B). We identified co-expressing neurons in all neuromers of the VNC, with the ventral location of the mesothoracic *aPKC/FoxP* neurons suggesting potential wing MNs ( Figure 2C). ^78^ To ascertain the identity of this ventral cluster of co-expressing neurons, we marked MNs with the driver line D42-Gal4 and used FoxP-LexA to stain all *FoxP* neurons. With these labels, we identified a ventral sub-population of putative wing MNs expressing *FoxP*, which matched the location of the *aPKC/FoxP* neurons identified before ( Figure 2D). The overlap (or lack thereof) may not be clearly visible in these 2D renderings presented in this text, which is why we made the 3D image stacks available for closer scrutiny (DOI: 10.5281/zenodo.10047941). ^79^ Both the C380 driver used in Figure 1 and the D42 driver used here, label not only MNs but also other neurons, in particular cholinergic neurons. However, the lines overlap in MNs in the VNC. *FoxP* is also expressed in these MNs and knocking out aPKC in these neurons also impaired operant self-learning. While each line of evidence is suggestive, at best, together, these lines of evidence converge on the hypothesis that wing steering MNs expressing both aPKC and FoxP may be important for operant self-learning. These suggestive results, together with the recently published draft VNC connectome, ^80^ motivated us to analyze the direct wing steering muscles for innervation by MNs with both aPKC and *FoxP* expression, instead of further quantifying the VNC dataset.

**Figure 3.**
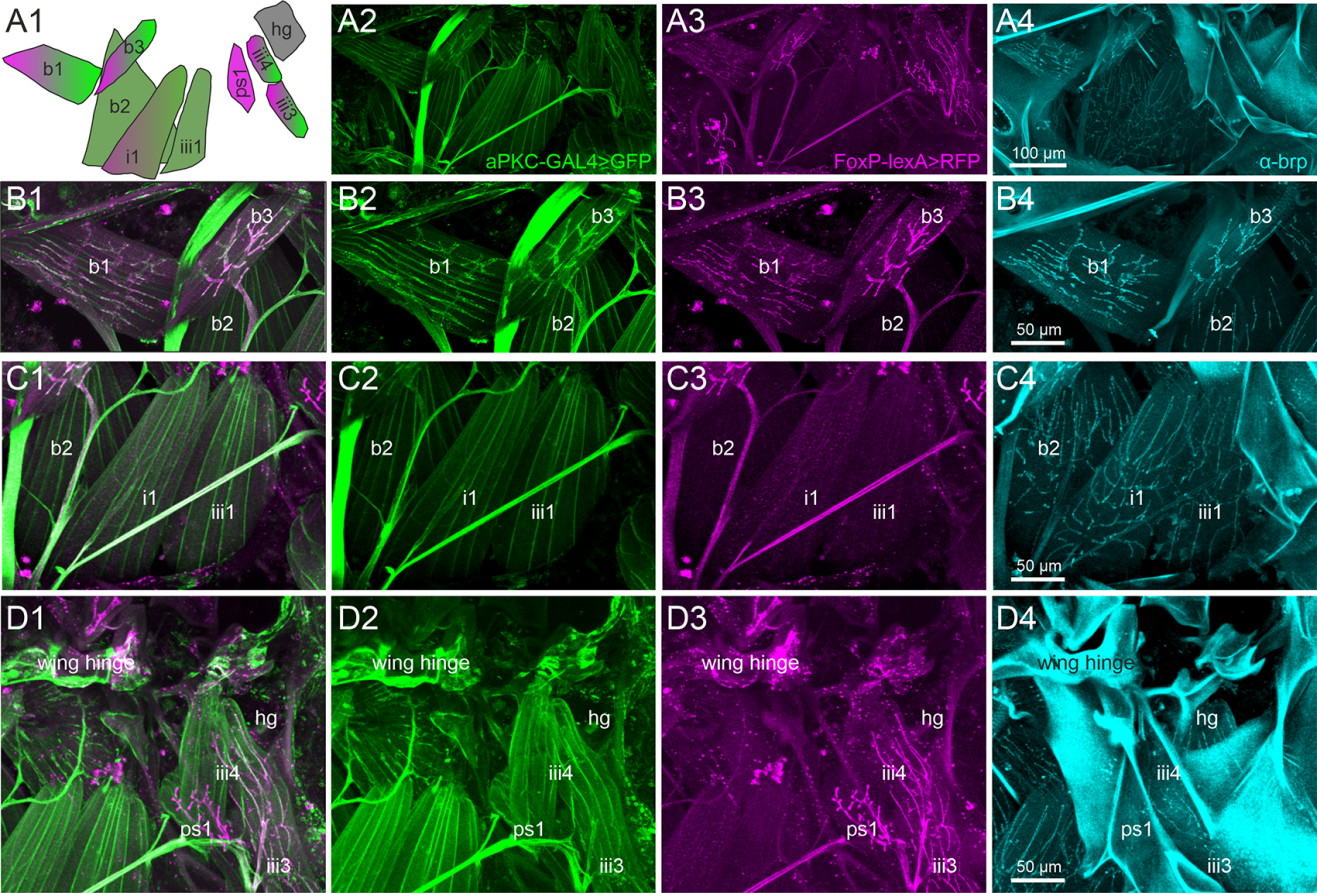
A subset of direct wing muscles is innervated by FoxP/aPKC co-expressing MNs. Representative projection view of MN terminals on the direct flight steering muscles in animals with GFP label in aPKC expressing cells (aPKC-Gal4>CD8::GFP, green), RFP expression in FoxP expressing cells (FoxP-LexA>CD8::RFP, magenta), and immunolabeling for the presynaptic active zone marker *bruchpilot* ( *brp*, cyan) reveal which direct flight steering MNs express either aPKC, FoxP, or both, or none of them but only *brp* in presynaptic active zones. (A1) depicts the orientation, shape, and abbreviated names of direct flight steering muscles and summarizes which ones are innervated by aPKC expressing MNs (green), by FoxP-expressing MNs (magenta), or by MNs without FoxP and aPKC expression (grey). (A2) Projection view of direct flight muscles and their innervation with GFP expression under the control of aPKC-GAL4 (green) at 20x magnification. (A3) Same preparation, image stack, and field of view but with RFP expression under the control of FoxP-lexA (magenta). (A4) Same preparation, image stack, and field of view with *brp* immunolabel (cyan) in presynaptic active zones of flight steering MNs. (B1-B4). Same preparation but with selective enlargement of the three basalare muscles (b1-b3), with all three labels in (B1), GFP label in aPKC-expressing cells (green, B2), RFP label in FoxP expressing cells (magenta, B3), and Brp label in presynaptic active zones (cyan, B4). Muscles b1 and b3 are innervated by steering MNs with aPKC and FoxP expression, but b2 is devoid of FoxP-expressing innervation. (C1-C4) Same preparation but with selective enlargement of second basalare (b2) and the adjacent pterale 1 (i1) and pterale II (iii3) muscles with all three labels (C1), GFP label in aPKC-expressing cells (green, C2), RFP label in FoxP-expressing cells (magenta, C3), and *brp* label in presynaptic active zones (cyan, C4). Only i1 is faintly labeled for terminals with aPKC FoxP expression. (D1-D4) Same preparation but with selective enlargement of the pterale II muscles iii3 and iii4, the adjacent pleurosternal muscle (ps1), and the posterior notal wing process muscles (hg) with all three labels (D1), GFP label in aPKC-expressing cells (green, D2), RFP label in FoxP-expressing cells (magenta, D3), and Brp label in presynaptic active zones (cyan, C4). The pterale II muscles iii3 and iii4 are innervated by terminals with aPKC and FoxP expression. Image stacks available at: 10.5281/zenodo.10606166.

Each direct steering muscle is innervated by one single identified MN. ^81^ Thus, identifying the muscles reveals the identity of the corresponding MNs. To test for the expression of aPKC and *FoxP* in MNs that innervate direct wing muscles, we expressed UAS-GFP under the control of aPKC-Gal4 ( Figure 3, second column) and RFP under the control of FoxP-lexA ( Figure 3, third column). The signal of each fluorescent reporter was further enhanced by immunocytochemistry, and the active zone marker *bruchpilot* ( *brp* ^66^; was used to label the neuromuscular synapses in flight MN axon terminals ( Figure 3, fourth column). In total, *Drosophila* is equipped with 12 flight steering muscles each of which likely contributes to a distinct function in flight control. ^82^ The activation patterns of four of these muscles have been analyzed in response to optomotor stimulation during flight (first and second basalar muscles b1 and b2, as well as first pterale I muscle (i1) and third pterale II muscle (iii1). ^82^^–^^84^ A schematic of these four direct flight muscles plus five adjacent ones that were analyzed in this study illustrates their spatial arrangement, shapes, and depicts which ones are innervated by steering MNs that express both aPKC (green) and FoxP (magenta), only one of both, or neither of them (grey; Figure 3A1).

Representative maximum intensities projection views at 20x magnification allow us to visualize all nine flight steering muscles investigated in one field of view ( Figures 3A2-A4). Except the pleurosternal muscle 1 (ps1) and the posterior notal wing process muscles (hg), all other steering muscles are innervated by aPKC-positive MNs ( Figures 3A1, A2, green). In contrast, six of the nine steering muscles are innervated by *FoxP*-positive MNs, including the basalars b1 and b3, the pterale i1, iii3, and iii4, as well as the pleurosternal muscle ps1 ( Figures 3A1, A3, magenta). A subset of five flight steering muscles is innervated by MNs that express both genes required for operant self-learning. These are b1, b3, i1, iii3, and iii4 ( Figure 3A).

Selective enlargements at 40x magnification were used to better visualize the axon terminals on those steering muscles of particular interest, with Figure 3 panels B1-B4 focussing on the three muscles that insert at the basalar sclerite and are thus named basalar 1-3 (b1-b3). Prominent labels of both aPKC (green) and *FoxP* (magenta) are present in the MNs to steering muscles b1 and b3, but the motor axon innervating b2 is devoid of *FoxP* signals ( Figure 3, B1-B4).

Muscle b1 has been reported to fire once every wingbeat ^83^ during straight flight, during the transition from up- to downstroke. Optomotor stimulation causes phase shifts of b1 firing in the wing beat cycle, which in turn cause changes in wing beat amplitude both in the fruit fly *Drosophila* ^83^ as well as in the blowfly, *Calliphora.* ^85^ The activity of b3 during flight has not been recorded electrophysiologically, but the muscle exhibits very similar morphological properties compared to b1. Both b1 and b3 are orientated similarly relative to the body axis and the wing hinge, and both are innervated by MNs with particularly large diameter axons ( Figures 3B1-B3) and particularly large active zones ( Figure 3B4), suggesting similar functional roles. Steering muscle b2 is also innervated by large diameter axons ( Figure 3C1) with large presynaptic active zones ( Figure 3C4). Although some aPKC reporter label is detected in the MN to b2 ( Figures 3C1, C2, C4), labeling intensity is considerably fainter than that in b1 and b3 MNs ( Figures 3B1-B3). Fainter reporter labeling indicates weaker aPKC-Gal4 expression. The b2 MN is devoid of the *FoxP* reporter label ( Figure 3C3). Although b2 has been reported to respond to optomotor stimulation, its phasic bursting responses correlate with rapid changes in wing beat amplitude as observed during body saccades. ^83^

Figure 3 panels C1-C4 move the field of view posteriorly and show the pterale 1 muscle i1 and the pterale 2 muscle iii1. Muscle i1 is innervated by a MN with faint aPKC ( Figure 3C2) and faint *FoxP* ( Figure 3C3) label. i1 has been reported to respond to optomotor stimulation, but its specific role in optomotor control remains largely unknown. ^83^ Steering muscle iii1 shows some faint aPKC signal ( Figure 3C2) but is devoid of any *FoxP* label ( Figure 3C3) and does not participate in optomotor flight control. ^83^

The roles in flight control of the remaining four steering muscles (ps1, iii3, iii4, and hg; Figures 3D1-D4) in *Drosophila* are not fully understood. However, iii3 and iii4 are innervated by MNs with aPKC ( Figure 3D2) and *FoxP* ( Figure 3D3) expression. Axon terminals on ps1 are *FoxP* positive ( Figure 3D3) but lack any aPKC label ( Figure 3D2). Active zones on steering muscle hg are visible through the *brp* label ( Figure 3D4) but the motor axon on hg is devoid of both aPKC ( Figure 3D2) and *FoxP* ( Figure 3D3).

### Self-learning breaks optomotor symmetry

**Figure 4.**
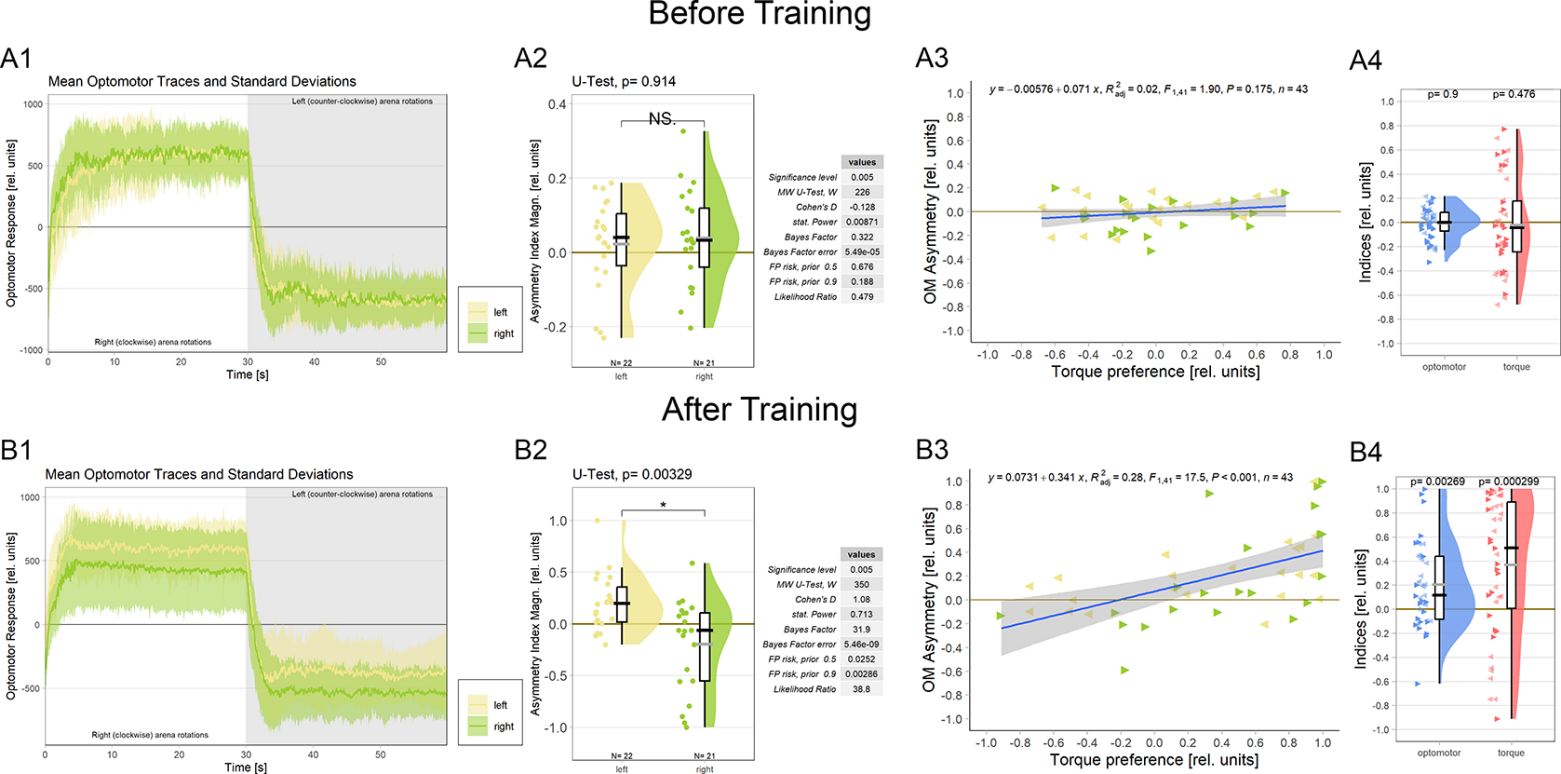
Associating one torque domain with heat changes optomotor behavior. A. Measurements before training. A1 Averaged optomotor traces of flies punished either on the ‘left’ (yellow) or on the ‘right’ (green) torque domain. Both groups show similar response magnitudes in either direction of the optomotor stimulus. Errors are standard deviations. A2 Optomotor asymmetry indices for flies punished either on the ‘left’ (yellow) or on the ‘right’ (green) torque domain. The values for both groups spread around zero. Positive values indicate a shift towards positive (right-turning) torque. Both frequentist and Bayesian analyses are displayed to the right of the plots and indicate no difference between the groups. A3 Regression analysis between torque preference and optomotor asymmetry. Optomotor values were adjusted such that positive values indicate a shift towards the unpunished torque domain. No significant correlation was observed. Left-pointing arrowheads (yellow) denote flies punished on left-turning torque and right-pointing arrowheads (green) denote flies punished on right-turning torque. A4 Comparison of optomotor asymmetry (left, yellow) and torque preference (right, green) indices. Here again, optomotor values were adjusted such that positive values indicate a shift towards the unpunished torque domain. Both measures vary around the zero point and Wilcoxon tests against zero are not significant (p-values above each plot). Left- and right-pointing arrowheads denote punishment directions as before. B. Measurements after training. B1 Averaged optomotor traces of flies punished either on the ‘left’ (yellow) or on the ‘right’ (green) torque domain. A reduction in the OMR magnitude can be observed on the punished, but not on the unpunished side. Errors are standard deviations. B2 Optomotor asymmetry indices for flies punished either on the ‘left’ (yellow) or on the ‘right’ (green) torque domain. Positive values indicate a shift towards positive (right-turning) torque. The values for each group have now shifted towards the unpunished side compared to the values before training. Both frequentist and Bayesian analyses are displayed to the right of the plots and indicate a significant difference between groups. B3 Regression analysis between torque preference and optomotor asymmetry. Optomotor values were adjusted such that positive values indicate a shift towards the unpunished torque domain. A significantly positive correlation was observed, such that higher torque preferences entailed larger optomotor asymmetry. Left-pointing arrowheads (yellow) denote flies punished on left-turning torque and right-pointing arrowheads (green) denote flies punished on right-turning torque. B4 Comparison of optomotor asymmetry (left, yellow) and torque preference (right, green) indices. Here again, optomotor values were adjusted such that positive values indicate a shift towards the unpunished torque domain. Both measures are shifted towards more positive values and Wilcoxon tests against zero are now significant for both variables (p-values above each plot). Left- and right-pointing arrowheads denote punishment directions as before. Data available at: 10.5283/epub.54804. ^86^

Because it is not known at which torque meter reading the fly actually generates zero angular momentum, before each torque learning experiment, it is crucial to set the average of the asymptotic left and right optomotor response (OMR) magnitudes to zero ( Figure 4A1, see Methods for details). This is to avoid an initial bias in the torque preference before training and with the assumption that the mid-point between the maximal OMRs roughly corresponds to flying straight (i.e, zero angular momentum). During the ensuing conditioning procedure, flies show spontaneous torque fluctuations that can reach or even exceed those elicited by optomotor stimulation in magnitude. Within each torque domain (i.e., ‘left’ or ‘right’, respectively), the temporal patterns (i.e., slow or fast) of torque fluctuations and their relative direction (i.e., more or less torque) are irrelevant for the heat stimulus as long as the zero point is not crossed: heat remains either on or off until the fly switches torque domains. Until this research, there was no reason to assume any relation between elicited OMRs and spontaneous torque fluctuations, neither conceptually nor anatomically. Now, however, the described role of the steering MNs co-expressing aPKC and FoxP ( Figure 3) in large torque fluctuations elicited by optomotor stimuli prompted the hypothesis that there may be a neuroanatomical connection between spontaneous torque fluctuations and elicited OMRs after all: the motor neurons that innervate the steering muscles may be involved in both elicited and spontaneous torque fluctuations. If these neurons were indeed common to elicited OMRs and spontaneous torque fluctuations, we should observe a change in the OMRs after operant yaw torque learning.

To test this hypothesis, we analyzed the OMRs after training of a cohort of wild type Berlin flies ( Figure 4B; control flies from a separate research project). We found that the asymptotic magnitude of the OMR on the punished side was reduced after operant training, while the OMR on the unpunished side remained unaltered compared to before training ( Figure 4B1). Quantifying this observation revealed a significant difference in optomotor asymmetry after training ( Figure 4B2) but not before training ( Figure 4A2) between the two experimental groups.

An important question regarding the functional significance of this change in optomotor asymmetry is whether the amount of torque preference after training reflects the amount of optomotor asymmetry. There was no significant correlation between torque preference and optomotor asymmetry before training ( Figure 4A3; as expected as the OMR was adjusted to be as symmetrical as practically possible). Once the flies have completed the training phase, torque preference becomes a significant predictor of optomotor asymmetry ( Figure 4B3). These results corroborate our hypothesis that the wing MNs identified above, specifically those that have been shown to be involved in generating OMRs, ^82^^,^^83^ constitute a site of the plasticity mediating operant self-learning in *Drosophila.* We also tested whether it would be sufficient to test for optomotor asymmetry after training as a proxy measure for torque preference. Before training, as expected, both measures showed similar values ranging around the zero point ( Figure 4A4). After training, both measures did deviate from zero towards the unpunished direction, however, the effect was noticeably larger for torque preference than for optomotor asymmetry ( Figure 4B4). Together with the correlation explaining about a third of the variance ( Figure 4B3), these data may indicate that the MNs are an important, but not the only site of plasticity in this form of learning.

### Spatial genome editing: *FoxP* likely not required in the brain

**Figure 5.**
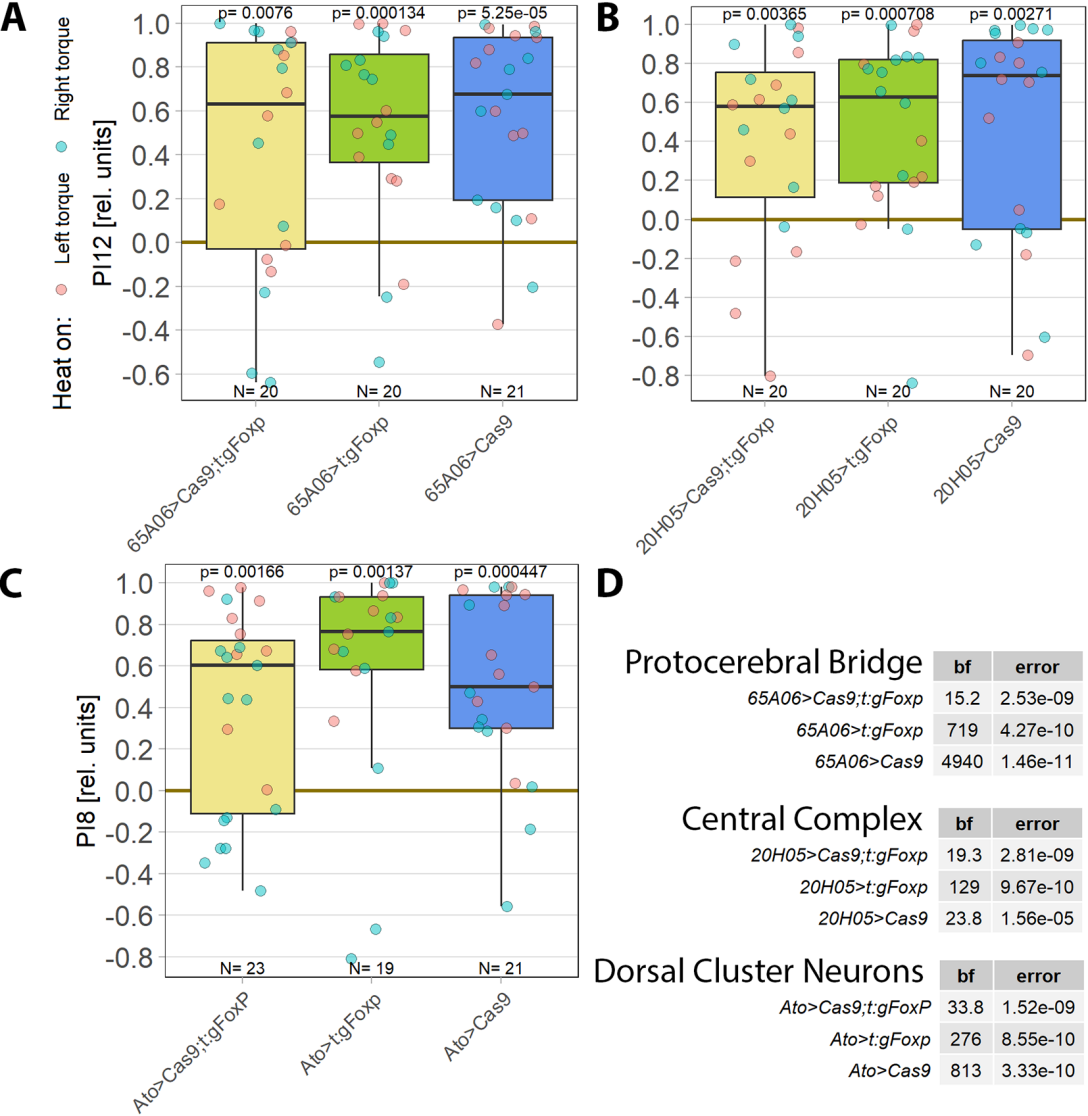
For operant self-learning, *FoxP* is likely not required in the brain. Performance indices for the first period after training (PI8/12) are plotted. Each colored dot represents a single fly experiment. Red dots denote experiments where the fly was punished on its left turning torque domain, blue dots denote flies that were punished on their right turning domain. Box plots denote medians, quartiles and non-outlier range. Statistical analyses test for differences of PIs against zero (above plots and D). A. Flies with *FoxP* knocked out in the protocerebral bridge (left, yellow) as well as the gRNA (middle, green) and Cas9 (right, blue) control flies showed high PIs, large Bayes factors (D) and small p-values, indicating their self-learning was intact. Data: 10.5283/epub.52956. ^88^ B. Flies with *FoxP* knocked out in the protocerebral bridge and additional components of the central complex (left. yellow) as well as the gRNA (middle, green) and Cas9 (right, blue) control flies showed high PIs, large Bayes factors (D) and small p-values, indicating their self-learning was intact. Data: 10.5283/epub.52951. ^89^ C. Flies with *FoxP* knocked out in the dorsal cluster neurons (left, yellow) as well as the gRNA (middle, green) and Cas9 (right, blue) control flies showed high PIs, large Bayes factors (D) and small p-values, indicating their self-learning was intact. Data: 10.5283/epub.52946. ^89^^,^^90^ D. Bayesian statistics for the three datasets.

Optomotor analysis ( Figure 4) suggests there may be additional sites of plasticity besides wing steering MNs. *FoxP*-positive neurons in the brain are straightforward potential candidates for such additional sites. Therefore, we performed spatial CRISPR/Cas9-based genome editing without restricting the manipulation to the adult stage. At the time of these experiments, no driver lines with *FoxP*-overlapping expression in the Saddle and Vest regions were available. Therefore, we tested driver lines expressing in the protocerebral bridge (PCB, Figure 5A), the PCB and adjacent central complex neuropils (see Materials and Methods for details, Figure 5B) and the dorsal cluster neurons ( Figure 5C). Overlap of Gal4 expression patterns with FoxP expression was verified using the FoxP-LexA line ^77^^,^^87^ and FoxP-knockout efficiency was quantified previously. ^77^ No self-learning impairment was observed following *FoxP* knockout in these brain areas, suggesting that *FoxP* expression is not necessary in these areas for operant self-learning.

We discovered that aPKC is required in MNs for operant self-learning (see above). As *FoxP* is also expressed in MNs, we knocked *FoxP* out in MNs using two different driver lines, C380 and D42. However, CRISPR/Cas9-mediated *FoxP* knockout in MNs disrupted flight-performance of manipulated flies to an extent that precluded any torque learning experiments. Our results above ( Figure 3) will now allow us to select more specific driver lines, expressing only in the identified wing steering MNs, ^91^ which may yield *FoxP* knock-out flies (or any other manipulation) with sufficient flight performance.

### Temporal genome editing: Self-learning impaired only after a 2-week *FoxP* knockout

**Figure 6.**
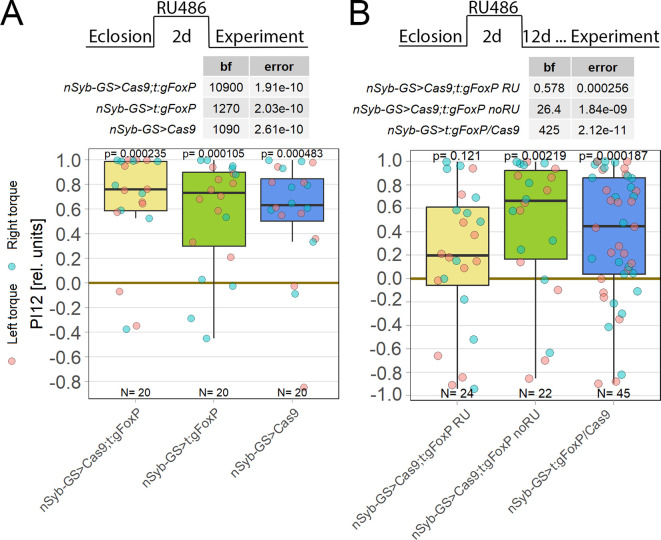
Knockout of *FoxP* in adult *Drosophila* shows learning impairments only after 14 days. Plotted are performance indices for the first period after training (PI12). Each colored dot represents a single fly experiment. Red dots denote experiments where the fly was punished on its left turning torque domain, blue dots denote flies that were punished on their right turning domain. Box plots denote medians, quartiles and non-outlier range. Statistical analyses test for differences of PIs against zero. A. Self-learning two days after *FoxP* knockout induction with RU486. Experimental animals (left, yellow) as well as gRNA (middle, green) and Cas9 (left, blue) control animals showed all high PIs as well as large Bayes Factors above 1000 and p-values below 0.005, indicating that all groups showed unimpaired self-learning. Data: 10.5283/epub.52963. ^93^ B. Self-learning 14 days after *FoxP* knockout induction with RU486 and 12 days after cessation of RU486 administration. Experimental animals (left, yellow) showed low PIs as well as a Bayes Factor of less than one together with a large p-value, whereas both the genetic control animals without RU486 treatment (middle, green) and the pooled RU486-treated gRNA and Cas9 controls (right, blue) showed high PIs, large Bayes Factors and p-values smaller than 0.005, indicating an impairment in self-learning only in the experimental group. Data: 10.5283/epub.52964. ^94^

*FoxP* was shown to be important for normal development. ^77^^,^^92^ Strong motor impairments have been reported after a developmental *FoxP* knockout, for instance rendering the animals unable to fly (see above). Since the ability to fly is a basic requirement for torque learning, we used a CRISPR/Cas9-based approach to pan-neuronally knock out *FoxP* in adult flies. No effect was observed on self-learning two days after the start of the knock-out induction ( Figure 6A), but waiting for 14 days after the pan-neural knock-out yielded a significant self-learning impairment ( Figure 6B). As a test seven days after induction was also without effect (DOI: 10.5283/epub.52965), self-learning remains functional for at least 7-14 days after cessation of *FoxP* gene transcription in all neurons.

### *aPKC* acts via non-canonical pathways in self-learning

**Figure 7.**
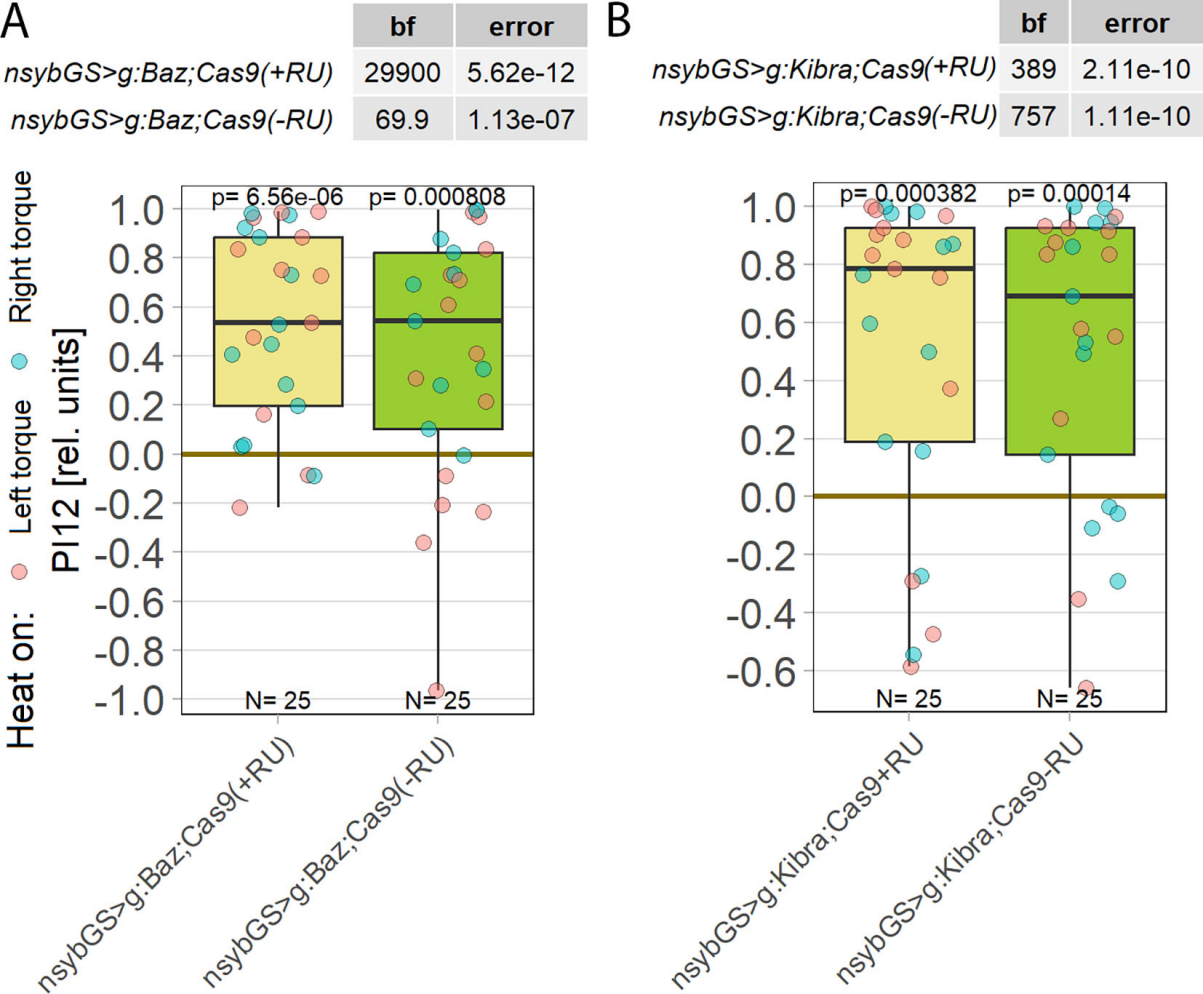
Neither *bazooka* nor KIBRA are involved in operant self-learning. Plotted are performance indices for the first period after training (PI12). Each colored dot represents a single fly experiment. Red dots denote experiments where the fly was punished on its left turning torque domain, blue dots denote flies that were punished on their right turning domain. Box plots denote medians, quartiles and non-outlier range. Statistical analyses test for differences of PIs against zero. A. Knocking out *bazooka* using CRISPR/Cas9-mediated genome editing in adult animals had no effect on operant self-learning. Both flies that were fed the steroid hormone RU486 (left, yellow) and the genetically identical flies without the hormone (right, green), showed high PIs, low p-values and high Bayes Factors, indicating a preference for the unpunished torque domain. Data: 10.5283/epub.52947. ^107^ B. Knocking out KIBRA using CRISPR/Cas9-mediated genome editing in adult animals had no effect on operant self-learning. Both flies that were fed the steroid hormone RU486 (left, yellow) and the genetically identical flies without the hormone (right, green), showed high PIs, low p-values and high Bayes Factors, indicating a preference for the unpunished torque domain. Data: 10.5283/epub.53685. ^108^

Having established that *aPKC* is required in *FoxP*-positive MNs, we sought to identify further components of the *aPKC*-dependent plasticity underlying operant self-learning in *Drosophila.* It is not uncommon for plasticity mechanisms in the adult animal to recruit genes with a function during neuronal development. ^95^^–^^97^ With PKCs being notorious for being able to compensate for genetic manipulations, ^69^^–^^73^ one reliable countermeasure has proven to shorten the time period between manipulation and testing sufficiently to ensure compensation has no time to take place. ^21^^,^^31^ Following this tried-and-tested approach, our manipulations of prominent PKC interaction partners were thus restricted to adult neurons. One such prominent interaction partner of *aPKC* during *Drosophila* nervous system development is *bazooka* ( *baz*), a crucial component of the highly conserved PAR complex. ^98^^,^^99^ However, knocking out *baz* in all adult neurons did not disrupt operant self-learning ( Figure 7A), suggesting that the Par complex signaling pathway is not involved in operant self-learning. A second prominent aPKC interaction partner is the kidney and brain protein (KIBRA), which acts in the conserved Hippo pathway ^100^^,^^101^ and also proposed to be involved in learning/memory. ^102^^–^^106^ Knocking out KIBRA in all adult neurons also did not disrupt operant self-learning ( Figure 7B).

## Discussion

Operant self-learning in *Drosophila* is a form of motor learning that appears to be conserved among bilaterians. The transcription factor *FoxP* is involved in various forms of motor learning in chordates, ^5^^–^^9^^,^^11^^,^^13^^,^^16^^–^^18^ as is PKC. ^22^^–^^24^^,^^109^^–^^112^ PKC is also involved in motor learning in the feeding behavior of the lophotrochozoan *Aplysia* ^113^ and both are involved in motor learning in the ecdysozoan *Drosophila* (this work and. ^20^^,^^21^^,^^31^ This wealth of evidence supports what has been called ‘deep homology’ for motor learning in bilaterians. ^12^

In this work, we present new insights into the neurobiological mechanisms underlying this form of motor learning.

### Self-learning requires *aPKC* in *FoxP* wing steering motor neurons

While previous evidence suggested that some PKC activity was required in some MNs for operant self-learning, ^21^ it was not clear which PKC gene was involved and in which MNs. Improving upon the previous attempts to identify the responsible PKC gene, here we used CRISPR/Cas9 to first rule out that *PKC53e* is involved and then show that the atypical PKC (aPKC) is necessary for operant self-learning ( Figure 1). Restricting transgenic manipulations to *FoxP* isoform-B positive neurons and using anatomical experiments to confirm behavioral results suggesting that *FoxP* is not necessary in the brain for operant self-learning ( Figures 2, 5), we now hypothesize that *aPKC* activity is required in aPKC/ *FoxP*-positive direct wing steering MNs in the ventral nerve cord (VNC). Our analysis of these direct wing muscles, responsible for, e.g. wing beat amplitude (a major contributing factor to yaw torque), ^114^ revealed *aPKC/FoxP* co-expressing MNs innervating a specific subset of these muscles ( Figure 3), corroborating our hypothesis.

### Motor neurons involved in optomotor responses co-express aPKC and *FoxP*

Specifically, the basalar muscle b1 which is known to regulate wing beat amplitude during optomotor stimulation through phase shifts of its action potential within the wing beat cycle ^83^ is innervated by a MN with very strong aPKC and *FoxP* expression in all 11 animals analyzed. Similarly, b3 is also innervated by a MN with very strong aPKC and *FoxP* expression and the b3 muscle is reported to increase activity when the ipsilateral wing decreases its amplitude. ^115^ The b1 and the b3 muscles thus both share similar sizes and morphologies, are innervated by MNs with particularly thick processes and presynaptic active zones, insert at opposite sides of the basalar sclerite and act in a push-pull fashion in controlling wing beat amplitude, ^115^ a major contributor to yaw torque. Although the MN innervating the third basalar muscle, b2, also expresses aPKC along the axon, we did not detect aPKC signal in axon terminals or active zones, and it does not show *FoxP* label, thus ruling out a function of b2 in aPKC/FoxP mediated operant self-learning. In contrast to b1 and b3, b2 firing is not linked to the wingbeat cycle; instead, it fires in bursts during turning maneuvers. ^82^ Corroborating the conclusion that it is not involved in mediating operant self-learning, b2 is silent during straight flight, but it is likely involved in regulating body saccades (Heide, Götz, 1996), which are by themselves not relevant for controlling the heat in our experiments. For the basalar muscles the emerging picture is that b1 and b3 are the top candidates for self-learning because they likely regulate wingbeat amplitude and thus also yaw torque on a wingbeat cycle by cycle basis. Strikingly, the MNs innervating these two muscles show high levels of aPKC and FoxP expression. Although much less is known about the pterale muscles, and neither iii3 nor iii4 (both with aPKC and FoxP co-expression) have been recorded electrophysiologically during flight, a similar picture begins to emerge. The MN to the left muscle i1 is known to be active during right turns ^83^ and co-expresses aPKC and FoxP, whereas iii1 reportedly ^83^ does not participate in OMRs and is innervated by a MN with weak aPKC and no *FoxP* label. In summary, flight steering muscles that are known to regulate flight steering in response to optomotor stimulation receive aPKC and *FoxP* positive motor innervation.

### Operant self-learning modifies optomotor responses

Given that MNs innervating muscles important for controlling wingbeat amplitudes during OMRs coexpress aPKC and *FoxP*, the genes necessary for our operant experiments where the animals learn to generate specific wingbeat amplitudes, a straightforward hypothesis emerges: if operant self-learning altered the properties of MNs that also happen to be involved in OMRs, we should be able to observe training-induced modifications in the flies’ optomotor behavior after training. Analyzing the OMRs of a separate cohort of wild-type flies corroborated this hypothesis ( Figure 4). The optomotor magnitude was reduced in the punished turning direction, but unaltered in the unpunished direction. This result is both expected and unexpected: it is expected because these MNs express both genes necessary for operant self-learning. The result is unexpected because OMRs were not trained and the magnitude of the effect is not large enough to prevent the flies from flying straight. ^30^ So unexpected was this result, in fact, that our study, triggered by the expression results ( Figures 2, 3), is the first to analyze OMRs after conditioning since the initial description of these operant experiments more than 30 years ago, ^35^ even though that capability existed from the very beginning. This surprising result from wild type flies raises the question of whether the changes observed in the OMR could have their cause outside of the steering MNs? With emerging evidence that OMRs are mediated via (both identified and yet to be identified) descending neurons from the brain with direct synaptic connections onto the steering MNs in the VNC, ^116^^,^^117^ additional neuroanatomical overlap between the circuitry controlling elicited OMRs and the circuitry giving rise to spontaneous yaw torque fluctuations becomes very unlikely. In particular, in the light of this evidence, any such overlap would need to be localized to the brain, where we did not find any neurons expressing both aPKC and FoxP. The remote possibility remains that OMR plasticity after operant learning may be caused by non-aPKC/Foxp-dependent plasticity mechanisms in the brain. Future research will address this possibility. Taken together, our results and the available evidence on OMRs strongly suggest that the changes we have detected in wild-type OMRs after operant self-learning are caused by steering MN plasticity, in turn brought about by the operant training.

Then again, only about a third of the variance in the torque preference after training can be explained by this optomotor asymmetry. This result explains the observations that there are flies with a strong conditioned torque preference but with an optomotor asymmetry in the opposite direction, as well as flies with a weak conditioned torque preference and large optomotor asymmetry ( Figure 4B3). Clearly, plasticity in steering MNs appears to be important, but it does not reflect the entirety of the learning processes.

Plasticity in steering movements such as OMRs have been observed before, such as in classic “inversion goggles” experiments where the coupling between the fly’s movements and the environment was reversed ^118^ or in a more recent experiment revealing adaptation processes. ^119^ It has long been recognized that insects with asymmetrical wing damage need to adjust the neural commands for generating torque to compensate for the changed physical torque (e.g., Refs. 116, 117, 120– 124). Plasticity in wing steering motor neurons provides a potential mechanism for such adjustments.

### Motoneuron plasticity mediates operant self-learning

Taken together, these results converge on the hypothesis that plasticity in MNs that innervate the direct muscles involved in generating yaw torque, but not other steering MNs, mediates an important aspect of operant self-learning in *Drosophila.* The importance of MN plasticity is emphasized by *FoxP*-dependent plasticity apparently not being required in the brain ( Figure 5). This MN plasticity could either be implemented by (a) postsynaptic plasticity of the input synapses to these MNs, postsynaptic because the MNs but not the interneurons express the proteins required for self-learning, or (b) on the level of the intrinsic excitability of flight steering motoneurons, or (c) on the level of the output synapses to the respective steering muscles. However, the mechanism and subcellular localization of self-learning in MNs remains to be determined.

A second interesting, yet unstudied population of *aPKC/FoxP* co-expressing neurons resides in the abdominal neuromer of the VNC. As flies use their abdomen analogously to a rudder during turns in flight, ^125^^,^^126^ involvement of these neurons seems plausible in addition to wing MNs.

While few studies in insects have shown MN plasticity, the *Aplysia* sensorimotor synapse is a classical model for research on plasticity mechanisms. There is a rich literature on MN plasticity in this preparation, some of which reports PKC-dependent mechanisms. ^104^^,^^127^^–^^137^ Also in mammals (including humans) MN plasticity in the spinal cord is a readily observable phenomenon in nonclinical and clinical settings. ^138^^–^^147^ The discovery of aPKC-dependent plasticity in *Drosophila* MNs expands this body of literature to a genetically tractable organism and inasmuch as clinical practice relies on MN plasticity, may even help instruct the development of clinical applications.

Until our work, PKC activity had only been shown to be important for memory consolidation/maintenance in world-learning experiments in flies, but not for learning/acquisition. ^148^^,^^149^ Also in *Drosophila* at the torque meter, PKCs are dispensable for world-learning. ^31^ The literature on PKCs in learning and memory in other animals is complex and multifaceted. In some preparations, PKC isoforms are required during memory maintenance, in some also during acquisition and in others different isoforms distinguish between acquisition and consolidation. ^69^^,^^70^^,^^102^^,^^104^^,^^128^^,^^133^^,^^135^^,^^150^^–^^158^ As the manipulations in our experiments lasted throughout training together with the tests immediately following training and we did not test for long-term memory, we can only ascertain that aPKC is required in a very narrow time window of minutes around training. Future research will address whether aPKC must be present during training, test, or both.

Notably, there is one other preparation where PKC activity is involved and which is also conceptually most closely resembling the one we used here, operant reward learning in *Aplysia* feeding behavior. However, in this preparation, the calcium-dependent Apl-I PKC not the atypical Apl-III PKC appears to be mediating the plasticity. ^113^ Given the degeneracy between the different PKC genes and the fact that they can not only compensate for long-term PKCi-mediated inhibition (this work and, ^21^ but also for each other, ^69^ more research is needed to elucidate how these different mechanisms of plasticity evolved and are related to each other.

### Self-learning may be mediated by a non-canonical *aPKC/FoxP* pathway

Neither *bazooka* ( *baz*) nor the kidney and brain gene (KIBRA), two prominent interaction partners of aPKC, ^98^^–^^104^^,^^106^^,^^159^^,^^160^ showed an effect on operant self-learning when they were knocked out in the adult nervous system ( Figure 7), potentially raising doubts about the effectiveness of the CRISPR/Cas9 method in these cases. In particular, one may question the approach of temporally limiting the manipulation to adult neurons. Although this had been effective with previous PKC manipulations using an inhibitory peptide, PKCi, ^21^^,^^31^ it only yielded an intermediate effect with a CRISPR-mediated knockout targeting aPKC ( Figure 1B). In contrast, temporally uncontrolled knockout of aPKC in MNs of the VNC ( Figure 1C) proved surprisingly effective, given expectations from prior experience.

Nonetheless, we are confident that both manipulations successfully knocked out each of these genes. First, we found that the *baz* knockout, rather than impairing self-learning, increases learning performance (manuscript in preparation), suggesting the PAR complex may be sequestering *aPKC* and thereby limiting its availability for self-learning. Thus, these data suggest that *baz* is indeed not directly involved in mediating the aPKC activity contributing to plasticity in steering MNs; instead, it may be binding aPKC in the PAR complex, preventing it from playing its role in MN plasticity. Second, our KIBRA knock-out had a severe effect on flight performance when elicited during development, suggesting that also this manipulation was, in principle, effective. That being said, without clear evidence that the *baz* and KIBRA proteins are completely absent, these results remain suggestive rather than conclusive. In addition to protein-level analysis of CRISPR efficacy (as we have performed in CRISPR-mediated FoxP knock-out ^77^), future experiments will use FoxP-iB and c380 drivers to drive baz and KIBRA knockouts.

Interestingly, at least during development, *baz* and KIBRA have been reported to have opposite effects on the function of *aPKC*, with the PAR complex ( *baz*) and the Hippo pathway (KIBRA) mutually inhibiting each other. ^161^ While *baz* is thought to mediate *aPKC* activity, KIBRA is thought to exert negative regulatory effects on *aPKC.* Thus, knocking out each one of them should have revealed a decrement in self-learning in at least one of them, if the processes during development were recapitulated during self-learning. On the other hand, experiments in which KIBRA has been shown to be involved in learning/memory have suggested a positive rather than a negative regulatory role, ^104^^,^^153^ albeit with an emphasis on long-term memory rather than learning. ^162^ Whichever way *aPKC* may be interacting with components of these canonical pathways, the literature predicts that at least one of our manipulations should have revealed a decrement in operant self-learning. The fact that this prediction was falsified may suggest that *aPKC* exerts its function in a non-canonical manner in operant self-learning plasticity. We are currently pursuing research into the possibility that *bazooka* may be a negative regulator of aPKC activity during operant self-learning.

### Persistent *FoxP* effect on operant self-learning in adults

As *FoxP* mutants are impaired in operant self-learning, ^20^ two hypotheses about the role of this prominent transcription factor arise. First, *FoxP* may be directly involved in the learning process via some unknown, cytosolic function. A transcription factor function appears unlikely, because of the short duration of our experiments. Second, *FoxP* may exert its effects as a developmental regulator, being crucial for the development of the circuits mediating operant self-learning. As the developmental role of FoxP genes is well documented ^7^^,^^14^^,^^77^^,^^92^^,^^163^^,^^164^ and there are few domains in the gene that would lend themselves to a hypothetical cytosolic function, the latter hypothesis appeared more plausible. The result that adult knockout of *FoxP* had no immediate effect on operant self-learning ( Figure 6A) seemed to corroborate this hypothesis. In contrast, supporting a continued role of FoxP genes in motor learning also after development are data from songbirds where *FoxP2* gene expression is not only regulated by singing, ^15^^,^^165^^–^^167^ but where normal *FoxP2* expression is necessary in adults to maintain learned song. ^8^^,^^168^ To also test the second hypothesis, we aged the flies after the *FoxP* knockout and tested them 7 and 14-days later. Flies still showed operant self-learning after seven days without *FoxP* transcription (DOI: 10.5283/epub.52965), but were impaired after 14 days ( Figure 6B). These results suggest a role for adult *FoxP* expression in maintaining operant self-learning capabilities after development, analogous to the role of FoxP2 in songbird vocal learning. ^8^^,^^168^ This result may be explained by the temporal dynamics of the genes regulated by *FoxP* ^169^ or by the half-life of FoxP itself. Further research is needed to distinguish between these two options.

## Data Availability

Underlying data for ‘Wings of Change: aPKC/FoxP-dependent plasticity in steering motor neurons underlies operant self-learning in *Drosophila*’, University of Regensburg: 1A: https://www.doi.org/10.5283/epub.52958 ^74^ University of Regensburg: 1B: https://www.doi.org/10.5283/epub.52957 ^75^ University of Regensburg: 1C: https://www.doi.org/10.5283/epub.52944 ^76^ Zenodo: 2: https://www.doi.org/10.5281/zenodo.10047941 ^79^ Zenodo: 3: https://www.doi.org/10.5281/zenodo.10606166 ^68^ University of Regensburg: 4: https://www.doi.org/10.5283/epub.54804 ^86^ University of Regensburg: 5A: https://www.doi.org/10.5283/epub.52956 ^88^ University of Regensburg: 5B: https://www.doi.org/10.5283/epub.52951 ^89^ University of Regensburg: 5C: https://www.doi.org/10.5283/epub.52946 ^90^ University of Regensburg: 6A: https://www.doi.org/10.5283/epub.52963 ^93^ University of Regensburg: 6B: https://www.doi.org/10.5283/epub.52964 ^94^ University of Regensburg: 7A: https://www.doi.org/10.5283/epub.52947 ^107^ University of Regensburg: 7B: https://www.doi.org/10.5283/epub.53685 ^108^ Data are available under the terms of the Creative Commons Zero “No rights reserved” data waiver (CC0 1.0 Public domain dedication).

## Software availability

Latest source code available from: https://github.com/brembslab/DTSevaluations

Archived software available from:

Software controlling experiments and collecting data: https://www.doi.org/10.5281/zenodo.7102195

Software evaluating data and data model: https://www.doi.org/10.5281/zenodo.10041052 ^39^

Python script keeping local and public datasets synchronized: https://www.doi.org/10.5281/zenodo.7101741

License: GPL-3.0

## References

[1] Arendt D : Elementary nervous systems. *Philos. Trans. R. Soc. Lond. Ser. B Biol. Sci* 2021 Mar 29;376(1821):20200347. 10.1098/rstb.2020.0347 33550948 PMC7935009

[2] Jékely G : The chemical brain hypothesis for the origin of nervous systems. *Philos. Trans. R. Soc. Lond. Ser. B Biol. Sci.* 2021 Mar 29;376(1821):20190761. 10.1098/rstb.2019.0761 33550946 PMC7935135

[3] Keijzer F Duijn M van Lyon P : What nervous systems do: early evolution, input–output, and the skin brain thesis. *Adapt. Behav.* 2013 Apr;21(2):67–85. 10.1177/1059712312465330

[4] Walsh B Smith A Weber-Fox C : Short-term plasticity in children’s speech motor systems. *Dev. Psychobiol.* 2006 Dec;48(8):660–674. 10.1002/dev.20185 17111401

[5] Lai CS Fisher SE Hurst JA : A forkhead-domain gene is mutated in a severe speech and language disorder. *Nature.* 2001 Oct 4;413(6855):519–523. 10.1038/35097076 11586359

[6] Bolhuis JJ Okanoya K Scharff C : Twitter evolution: converging mechanisms in birdsong and human speech. *Nat. Rev. Neurosci.* 2010 Nov;11(11):747–759. 10.1038/nrn2931 20959859

[7] Co M Anderson AG Konopka G : FOXP transcription factors in vertebrate brain development, function, and disorders. *Wiley Interdiscip. Rev. Dev. Biol.* 2020 Sep;9(5):e375. 10.1002/wdev.375 31999079 PMC8286808

[8] Day NF Hobbs TG Heston JB : Beyond Critical Period Learning: Striatal FoxP2 Affects the Active Maintenance of Learned Vocalizations in Adulthood. *eNeuro.* 2019 Mar;6(2):ENEURO.0071–ENEU19.2019. 10.1523/ENEURO.0071-19.2019 31001575 PMC6469881

[9] Fisher SE Scharff C : FOXP2 as a molecular window into speech and language. *Trends Genet.* 2009 Apr;25(4):166–177. 10.1016/j.tig.2009.03.002 19304338

[10] Zhang J Webb DM Podlaha O : Accelerated protein evolution and origins of human-specific features: Foxp2 as an example. *Genetics.* 2002 Dec;162(4):1825–1835. 10.1093/genetics/162.4.1825 12524352 PMC1462353

[11] Haesler S Rochefort C Georgi B : Incomplete and inaccurate vocal imitation after knockdown of FoxP2 in songbird basal ganglia nucleus Area X. *PLoS Biol.* 2007 Dec;5(12):e321. 10.1371/journal.pbio.0050321 18052609 PMC2100148

[12] Scharff C Petri J : Evo-devo, deep homology and FoxP2: implications for the evolution of speech and language. *Philos. Trans. R. Soc. Lond. Ser. B Biol. Sci.* 2011 Jul 27;366(1574):2124–2140. 10.1098/rstb.2011.0001 21690130 PMC3130369

[13] French CA Jin X Campbell TG : An aetiological Foxp2 mutation causes aberrant striatal activity and alters plasticity during skill learning. *Mol. Psychiatry.* 2012 Nov;17(11):1077–1085. 10.1038/mp.2011.105 21876543 PMC3481071

[14] Takahashi H Takahashi K Liu FC : FOXP genes, neural development, speech and language disorders. *Adv. Exp. Med. Biol.* 2009;665:117–129. 10.1007/978-1-4419-1599-3_9 20429420

[15] Teramitsu I Poopatanapong A Torrisi S : Striatal FoxP2 is actively regulated during songbird sensorimotor learning. *PLoS One.* 2010 Jan 6;5(1):e8548. 10.1371/journal.pone.0008548 20062527 PMC2796720

[16] French CA Vinueza Veloz MF Zhou K : Differential effects of Foxp2 disruption in distinct motor circuits. *Mol. Psychiatry.* 2019 Mar;24(3):447–462. 10.1038/s41380-018-0199-x 30108312 PMC6514880

[17] Groszer M Keays DA Deacon RMJ : Impaired synaptic plasticity and motor learning in mice with a point mutation implicated in human speech deficits. *Curr. Biol.* 2008 Mar 11;18(5):354–362. 10.1016/j.cub.2008.01.060 18328704 PMC2917768

[18] Schreiweis C Bornschein U Burguière E : Humanized Foxp2 accelerates learning by enhancing transitions from declarative to procedural performance. *Proc. Natl. Acad. Sci. USA.* 2014 Sep 30;111(39):14253–14258. 10.1073/pnas.1414542111 25225386 PMC4191787

[19] Lozano R Gbekie C Siper PM : FOXP1 syndrome: a review of the literature and practice parameters for medical assessment and monitoring. *J. Neurodev. Disord.* 2021 Apr 23;13(1):18. 10.1186/s11689-021-09358-1 33892622 PMC8066957

[20] Mendoza E Colomb J Rybak J : Drosophila FoxP mutants are deficient in operant self-learning. *PLoS One.* 2014 Jun 25;9(6):e100648. 10.1371/journal.pone.0100648 24964149 PMC4070984

[21] Colomb J Brembs B : PKC in motorneurons underlies self-learning, a form of motor learning in Drosophila. *PeerJ.* 2016 Apr 25;4:e1971. 10.7717/peerj.1971 27168980 PMC4860329

[22] Sakaguchi H Yamaguchi A : Early song-deprivation affects the expression of protein kinase C in the song control nuclei of the zebra finch during a sensitive period of song learning. *Neuroreport.* 1997 Aug 18;8(12):2645–2650. 10.1097/00001756-199708180-00002 9295093

[23] Metzger F Kapfhammer JP : Protein kinase C: its role in activity-dependent Purkinje cell dendritic development and plasticity. *Cerebellum.* 2003;2(3):206–214. 10.1080/14734220310016150 14509570

[24] Gallimore AR Kim T Tanaka-Yamamoto K : Switching On Depression and Potentiation in the Cerebellum. *Cell Rep.* 2018 Jan 16;22(3):722–733. 10.1016/j.celrep.2017.12.084 29346769

[25] Rochefort C Arabo A André M : Cerebellum shapes hippocampal spatial code. *Science.* 2011 Oct 21;334(6054):385–389. 10.1126/science.1207403 22021859

[26] Fresco N Tzelgov J Shmuelof L : How can caching explain automaticity? *Psychon. Bull. Rev.* 2022 Oct 12;30:407–420. 10.3758/s13423-022-02191-0 36224462

[27] Krakauer JW Hadjiosif AM Xu J : Motor Learning. *Compr. Physiol.* 2019 Mar 14;9(2):613–663. 10.1002/cphy.c170043 30873583

[28] Salmon DP Butters N : Neurobiology of skill and habit learning. *Curr. Opin. Neurobiol.* 1995 Apr;5(2):184–190. 10.1016/0959-4388(95)80025-5 7620306

[29] Du Y Krakauer JW Haith AM : The relationship between habits and motor skills in humans. *Trends Cogn. Sci.* 2022 May;26(5):371–387. 10.1016/j.tics.2022.02.002 35307293

[30] Brembs B : Mushroom bodies regulate habit formation in Drosophila. *Curr. Biol.* 2009 Aug 25;19(16):1351–1355. 10.1016/j.cub.2009.06.014 19576773

[31] Brembs B Plendl W : Double dissociation of PKC and AC manipulations on operant and classical learning in Drosophila. *Curr. Biol.* 2008 Aug 5;18(15):1168–1171. 10.1016/j.cub.2008.07.041 18674907

[32] Colomb J Brembs B : The biology of psychology: “Simple” conditioning? *Commun. Integr. Biol.* 2010 Mar;3(2):142–145. 10.4161/cib.3.2.10334 20585506 PMC2889970

[33] Brembs B : Spontaneous decisions and operant conditioning in fruit flies. *Behav. Process.* 2011 May;87(1):157–164. 10.1016/j.beproc.2011.02.005 21392558

[34] Götz KG : Optomoter studies of the visual system of several eye mutants of the fruit fly Drosophila. *Kybernetik.* 1964 Jun;2(2):77–92. 10.1007/BF00288561 5833196

[35] Wolf R Heisenberg M : Basic organization of operant behavior as revealed in Drosophila flight orientation. *J. Comp. Physiol. A.* 1991 Dec;169(6):699–705. 10.1007/BF00194898 1795235

[36] Guo A Li L Xia SZ : Conditioned visual flight orientation in Drosophila: dependence on age, practice, and diet. *Learn. Mem.* 1996 Jul-Aug;3(1):49–59. 10.1101/lm.3.1.49 10456076

[37] Brembs B : Operant learning of Drosophila at the torque meter. *J. Vis. Exp.* 2008 Jun 16;16. 10.3791/731 PMC2956216 19066552

[38] Tang S Juusola M : Intrinsic activity in the fly brain gates visual information during behavioral choices. *PLoS One.* 2010 Dec 30;5(12):e14455. 10.1371/journal.pone.0014455 21209935 PMC3012687

[39] Brembs B SimonBnm AnEhw : brembslab/DTSevaluations: Second public release. *Zenodo.* 2023. 10.5281/zenodo.10041052

[40] Ioannidis JPA : Why most published research findings are false. *PLoS Med.* 2005 Aug;2(8):e124. 10.1371/journal.pmed.0020124 16060722 PMC1182327

[41] Berrar D Dubitzky W : Should significance testing be abandoned in machine learning? *Int. J. Data Sci. Anal.* 2019 Jun;7(4):247–257. 10.1007/s41060-018-0148-4

[42] Berner D Amrhein V : Why and how we should join the shift from significance testing to estimation. *J. Evol. Biol.* 2022 Jun;35(6):777–787. 10.1111/jeb.14009 35582935 PMC9322409

[43] Maier M Lakens D : Justify your alpha: A primer on two practical approaches. *Adv. Methods Pract. Psychol. Sci.* 2022 Apr;5(2):251524592210803. 10.1177/25152459221080396

[44] Wulff JN Taylor L : EXPRESS: How and why alpha should depend on sample size: A Bayesian-frequentist compromise for significance testing. *Strateg. Organ.* 2023 Nov 6. 10.1177/14761270231214429

[45] Miller J Ulrich R : The quest for an optimal alpha. *PLoS One.* 2019 Jan 2;14(1):e0208631. 10.1371/journal.pone.0208631 30601826 PMC6314595

[46] Benjamin DJ Berger JO Johannesson M : Redefine statistical significance. *Nat. Hum. Behav.* 2018 Jan;2(1):6–10. 10.1038/s41562-017-0189-z 30980045

[47] Trafimow D Amrhein V Areshenkoff CN : Manipulating the Alpha Level Cannot Cure Significance Testing. *Front. Psychol.* 2018 May 15;9:699. 10.3389/fpsyg.2018.00699 29867666 PMC5962803

[48] Wasserstein RL Schirm AL Lazar NA : Moving to a world beyond “ *p* < 0.05.”. *Am. Stat.* 2019 Mar 29;73(sup1):1–19. 10.1080/00031305.2019.1583913

[49] Amrhein V Greenland S McShane B : Scientists rise up against statistical significance. *Nature.* 2019 Mar;567(7748):305–307. 10.1038/d41586-019-00857-9 30894741

[50] Ioannidis JPA : Retiring statistical significance would give bias a free pass. *Nature.* 2019 Mar;567(7749):461. 10.1038/d41586-019-00969-2 30903096

[51] Lakens D Adolfi FG Albers CJ : Justify your alpha. *Nat. Hum. Behav.* 2018 Feb 26;2(3):168–171. 10.1038/s41562-018-0311-x

[52] Gigerenzer G : Statistical rituals: The replication delusion and how we got there. *Adv. Methods Pract. Psychol. Sci.* 2018 Jun;1(2):198–218. 10.1177/2515245918771329

[53] Wasserstein RL Lazar NA : The ASA statement on p-values: Context, process, and purpose. *Am. Stat.* 2016 Apr 2;70(2):129–133. 10.1080/00031305.2016.1154108

[54] Colquhoun D : An investigation of the false discovery rate and the misinterpretation of p-values. *R. Soc. Open Sci.* 2014 Nov;1(3):140216. 10.1098/rsos.140216 26064558 PMC4448847

[55] Wagenmakers EJ Lee M Lodewyckx T : Bayesian Versus Frequentist Inference. *Bayesian Evaluation of Informative Hypotheses.* New York, NY: Springer New York;2008; pp.181–207. 10.1007/978-0-387-09612-4_9

[56] Wagenmakers EJ Love J Marsman M : Bayesian inference for psychology. Part II: Example applications with JASP. *Psychon. Bull. Rev.* 2018 Feb;25(1):58–76. 10.3758/s13423-017-1323-7 28685272 PMC5862926

[57] Wagenmakers EJ Marsman M Jamil T : Bayesian inference for psychology. Part I: Theoretical advantages and practical ramifications. *Psychon. Bull. Rev.* 2018 Feb;25(1):35–57. 10.3758/s13423-017-1343-3 28779455 PMC5862936

[58] Wagenmakers EJ Morey RD Lee MD : Bayesian benefits for the pragmatic researcher. *Curr. Dir. Psychol. Sci.* 2016 Jun;25(3):169–176. 10.1177/0963721416643289

[59] Malone HE Coyne I : Complementing the P-value from null-hypothesis significance testing with a Bayes factor from null-hypothesis Bayesian testing. *Nurse Res.* 2020 Nov 4;28:41–48. 10.7748/nr.2020.e1756 33150776

[60] Wei Z Yang A Rocha L : A Review of Bayesian Hypothesis Testing and Its Practical Implementations. *Entropy.* 2022 Jan 21;24(2). 10.3390/e24020161 35205456 PMC8871131

[61] Tendeiro JN Kiers HAL : A review of issues about null hypothesis Bayesian testing. *Psychol. Methods.* 2019 Dec;24(6):774–795. 10.1037/met0000221 31094544

[62] Brembs B Wiener J : Context and occasion setting in Drosophila visual learning. *Learn. Mem.* 2006 Sep-Oct;13(5):618–628. 10.1101/lm.318606 17015858 PMC1783616

[63] Liu L Wolf R Ernst R : Context generalization in Drosophila visual learning requires the mushroom bodies. *Nature.* 1999 Aug 19;400(6746):753–756. 10.1038/23456 10466722

[64] Liu G Seiler H Wen A : Distinct memory traces for two visual features in the Drosophila brain. *Nature.* 2006 Feb 2;439(7076):551–556. 10.1038/nature04381 16452971

[65] Brembs B Hempel de Ibarra N : Different parameters support generalization and discrimination learning in Drosophila at the flight simulator. *Learn. Mem.* 2006 Sep-Oct;13(5):629–637. 10.1101/lm.319406 17015859 PMC1783617

[66] Kittel RJ Wichmann C Rasse TM : Bruchpilot promotes active zone assembly, Ca2+ channel clustering, and vesicle release. *Science.* 2006 May 19;312(5776):1051–1054. 10.1126/science.1126308 16614170

[67] Vonhoff F Kuehn C Blumenstock S : Temporal coherency between receptor expression, neural activity and AP-1-dependent transcription regulates Drosophila motoneuron dendrite development. *Development.* 2013 Feb 1;140(3):606–616. 10.1242/dev.089235 23293292 PMC3561790

[68] Duch C : Original CLSM image stack for figure 3 for. *Zenodo.* 2024. 10.5281/zenodo.10606166

[69] Tsokas P Hsieh C Yao Y : Compensation for PKMζ in long-term potentiation and spatial long-term memory in mutant mice. *elife.* 2016 May 17;5. 10.7554/eLife.14846 27187150 PMC4869915

[70] Sacktor TC Hell JW : The genetics of PKMζ and memory maintenance. *Sci. Signal.* 2017 Nov 14;10(505). 10.1126/scisignal.aao2327 29138296 PMC6171341

[71] Teegala LR Elshoweikh Y Gudneppanavar R : Protein Kinase C α and β compensate for each other to promote stem cell factor-mediated KIT phosphorylation, mast cell viability and proliferation. *FASEB J.* 2022 May;36(5):e22273. 10.1096/fj.202101838RRR 35349200 PMC9298465

[72] Hiroki S Iino Y : The redundancy and diversity between two novel PKC isotypes that regulate learning in. *Proc. Natl. Acad. Sci. USA.* 2022 Jan 18;119(3). 10.1073/pnas.2106974119 35027448 PMC8784152

[73] Trzesniewski J Altmann S Jäger L : Reduced Purkinje cell size is compatible with near normal morphology and function of the cerebellar cortex in a mouse model of spinocerebellar ataxia. *Exp. Neurol.* 2019 Jan;311:205–212. 10.1016/j.expneurol.2018.10.004 30312605

[74] Ehweiner A : *PKCi expression in all or FoxPiB postive neurons.* Universität Regensburg;2022. Reference Source

[75] Ehweiner A : *Knockout of aPKC or PKC53e.* Universität Regensburg;2022. Reference Source

[76] Ehweiner A : *aPKC knockout.* Universität Regensburg;2022. Reference Source

[77] Palazzo O Rass M Brembs B : Identification of circuits involved in locomotion and object fixation in. *Open Biol.* 2020 Dec;10(12):200295. 10.1098/rsob.200295 33321059 PMC7776582

[78] Phelps JS Hildebrand DGC Graham BJ : Reconstruction of motor control circuits in adult Drosophila using automated transmission electron microscopy. *Cell.* 2021 Feb 4;184(3):759–774.e18. 10.1016/j.cell.2020.12.013 33400916 PMC8312698

[79] Ehweiner A : Confocal image stack of aPKC/FoxP co-staining. *Zenodo.* 2023. 10.5281/zenodo.10047941

[80] Azevedo A Lesser E Mark B : Tools for comprehensive reconstruction and analysis of *Drosophila*motor circuits. *bioRxiv.* 2022. 10.1101/2022.12.15.520299

[81] Trimarchi JR Schneiderman AM : The motor neurons innervating the direct flight muscles of Drosophila melanogaster are morphologically specialized. *J. Comp. Neurol.* 1994 Feb 15;340(3):427–443. 10.1002/cne.903400311 8188860

[82] Whitehead SC Leone S Lindsay T : Neuromuscular embodiment of feedback control elements in flight. *Sci. Adv.* 2022 Dec 14;8(50):eabo7461. 10.1126/sciadv.abo7461 36516241 PMC9750141

[83] Heide G Götz KG : Optomotor control of course and altitude in Drosophila melanogaster is correlated with distinct activities of at least three pairs of flight steering muscles. *J. Exp. Biol.* 1996 Aug;199(Pt 8):1711–1726. 10.1242/jeb.199.8.1711 8708578

[84] Dickinson MH Tu MS : The function of dipteran flight muscle. *Comp. Biochem. Physiol. A Comp. Physiol.* 1997 Mar;116(3):223–238. 10.1016/S0300-9629(96)00162-4

[85] Tu MS Dickinson MH : The control of wing kinematics by two steering muscles of the blowfly (Calliphora vicina). *J. Comp. Physiol. A.* 1996 Jun;178(6):813–830. 10.1007/BF00225830 8667294

[86] Brembs B : *Wild Type Berlin Torque Learning, left+right.* Universität Regensburg;2023. Reference Source

[87] Ehweiner A : *The neuronal basis of operant self-learning in Drosophila melanogaster.* Universität Regensburg;2022. Reference Source

[88] Ehweiner A : *GMR65A06-Gal4 x Cas9gFoxP.* Universität Regensburg;2022. Reference Source

[89] Ehweiner A : *GMR20H05-Gal4 x Cas9gFoxP.* Universität Regensburg;2022. Reference Source

[90] Ehweiner A : *Ato x Cas9gFoxP.* Universität Regensburg;2022. Reference Source

[91] Ehrhardt E Whitehead SC Namiki S : Single-cell type analysis of wing premotor circuits in the ventral nerve cord of. *bioRxiv.* 2023 Jun 1. 10.1101/2023.05.31.542897 37398009 PMC10312520

[92] Castells-Nobau A Eidhof I Fenckova M : Conserved regulation of neurodevelopmental processes and behavior by FoxP in Drosophila. *PLoS One.* 2019 Feb 12;14(2):e0211652. 10.1371/journal.pone.0211652 30753188 PMC6372147

[93] Ehweiner A : *gen switch (nSyb) adult Foxp knockout.* Universität Regensburg;2022. Reference Source

[94] Ehweiner A : *nsyb-GS adult Foxp knockout, test after 14 days.* Universität Regensburg;2022. Reference Source

[95] Leslie JH Nedivi E : Activity-regulated genes as mediators of neural circuit plasticity. *Prog. Neurobiol.* 2011 Aug;94(3):223–237. 10.1016/j.pneurobio.2011.05.002 21601615 PMC3134580

[96] Mayford M Kandel ER : Genetic approaches to memory storage. *Trends Genet.* 1999 Nov;15(11):463–470. 10.1016/S0168-9525(99)01846-6 10529810

[97] Greenspan RJ : Flies, genes, learning, and memory. *Neuron.* 1995 Oct;15(4):747–750. 10.1016/0896-6273(95)90165-5 7576623

[98] Pichaud F : PAR-Complex and Crumbs Function During Photoreceptor Morphogenesis and Retinal Degeneration. *Front. Cell. Neurosci.* 2018 Mar 29;12:90. 10.3389/fncel.2018.00090 29651238 PMC5884931

[99] Thompson BJ : Par-3 family proteins in cell polarity & adhesion. *FEBS J.* 2022 Feb;289(3):596–613. 10.1111/febs.15754 33565714 PMC9290619

[100] Yoshihama Y Chida K Ohno S : The KIBRA-aPKC connection: A potential regulator of membrane trafficking and cell polarity. *Commun. Integr. Biol.* 2012 Mar 1;5(2):146–151. 10.4161/cib.18849 22808318 PMC3376049

[101] Yoshihama Y Sasaki K Horikoshi Y : KIBRA suppresses apical exocytosis through inhibition of aPKC kinase activity in epithelial cells. *Curr. Biol.* 2011 Apr 26;21(8):705–711. 10.1016/j.cub.2011.03.029 21497093

[102] Sacktor TC : Memory maintenance by PKMζ--an evolutionary perspective. *Mol. Brain.* 2012 Sep 18;5:31. 10.1186/1756-6606-5-31 22986281 PMC3517905

[103] Heitz FD Farinelli M Mohanna S : The memory gene KIBRA is a bidirectional regulator of synaptic and structural plasticity in the adult brain. *Neurobiol. Learn. Mem.* 2016 Nov;135:100–114. 10.1016/j.nlm.2016.07.028 27498008

[104] Ferguson L Hu J Cai D : Isoform Specificity of PKMs during Long-Term Facilitation in Is Mediated through Stabilization by KIBRA. *J. Neurosci.* 2019 Oct 30;39(44):8632–8644. 10.1523/JNEUROSCI.0943-19.2019 31537706 PMC6820206

[105] Ji Z Li H Yang Z : Kibra Modulates Learning and Memory via Binding to Dendrin. *Cell Rep.* 2019 Feb 19;26(8):2064–77.e7. 10.1016/j.celrep.2019.01.097 30784589

[106] Zlomuzica A Preusser F Roberts S : The role of KIBRA in reconstructive episodic memory. *Mol. Med.* 2018 Mar 15;24(1):7. 10.1186/s10020-018-0007-8 30134813 PMC6016870

[107] Ehweiner A : *Baz knockout in all neurons.* Universität Regensburg;2022. Reference Source

[108] Ehweiner A : *gKibra adult 2g.* Universität Regensburg;2023. Reference Source

[109] Watanabe A Li R Kimura T : Lesions of an avian forebrain nucleus prevent changes in protein kinase C levels associated with deafening-induced vocal plasticity in adult songbirds. *Eur. J. Neurosci.* 2006 May;23(9):2447–2457. 10.1111/j.1460-9568.2006.04763.x 16706851

[110] Sakaguchi H : Effect of social factors on the development of PKC expression in songbird brain. *Neuroreport.* 2004 Dec 22;15(18):2819–2823. 15597061

[111] Yoshida Y Yamada T Sakaguchi H : Activation of protein kinase C by the error signal from a basal ganglia-forebrain circuit in the zebra finch song control nuclei. *Neuroreport.* 2003 Mar 24;14(4):645–649. 10.1097/00001756-200303240-00025 12657904

[112] Watanabe A Kimura T Sakaguchi H : Expression of protein kinase C in song control nuclei of deafened adult male Bengalese finches. *Neuroreport.* 2002 Jan 21;13(1):127–132. 10.1097/00001756-200201210-00030 11924874

[113] Lorenzetti FD Baxter DA Byrne JH : Molecular mechanisms underlying a cellular analog of operant reward learning. *Neuron.* 2008 Sep 11;59(5):815–828. 10.1016/j.neuron.2008.07.019 18786364 PMC2603610

[114] Dickinson MH : The initiation and control of rapid flight maneuvers in fruit flies. *Integr. Comp. Biol.* 2005 Apr;45(2):274–281. 10.1093/icb/45.2.274 21676771

[115] Lindsay T Sustar A Dickinson M : The Function and Organization of the Motor System Controlling Flight Maneuvers in Flies. *Curr. Biol.* 2017 Feb 6;27(3):345–358. 10.1016/j.cub.2016.12.018 28132816

[116] Namiki S Ros IG Morrow C : A population of descending neurons that regulates the flight motor of Drosophila. *Curr. Biol.* 2022 Mar 14;32(5):1189–96.e6. 10.1016/j.cub.2022.01.008 35090590 PMC9206711

[117] Palmer EH Omoto JJ Dickinson MH : The role of a population of descending neurons in the optomotor response in flying *Drosophila.* *bioRxiv.* 2022. 10.1101/2022.12.05.519224

[118] Wolf R Heisenberg M : Visual orientation in motion-blind flies is an operant behaviour. *Nature.* 1986 Sep;323(6084):154–156. 10.1038/323154a0

[119] Mano O Choi M Tanaka R : Long-timescale anti-directional rotation in optomotor behavior. *elife.* 2023 Sep 26;12. 10.7554/eLife.86076 37751469 PMC10522332

[120] Heisenberg M Wolf R : The sensory-motor link in motion-dependent flight control of flies. *Rev. Oculomot. Res.* 1993;5:265–283. 8420552

[121] Kassner Z Dafni E Ribak G : Kinematic compensation for wing loss in flying damselflies. *J. Insect Physiol.* 2016 Feb;85:1–9. 10.1016/j.jinsphys.2015.11.009 26598807

[122] Vance JT Roberts SP : The effects of artificial wing wear on the flight capacity of the honey bee Apis mellifera. *J. Insect Physiol.* 2014 Jun;65:27–36. 10.1016/j.jinsphys.2014.04.003 24768843

[123] Haas CA Cartar RV : Robust flight performance of bumble bees with artificially induced wing wear. *Can. J. Zool.* 2008 Jul;86(7):668–675. 10.1139/Z08-034

[124] Fernández MJ Springthorpe D Hedrick TL : Neuromuscular and biomechanical compensation for wing asymmetry in insect hovering flight. *J. Exp. Biol.* 2012 Oct 15;215(Pt 20):3631–3638. 10.1242/jeb.073627 22771747

[125] Zanker JM : How does lateral abdomen deflection contribute to flight control ofDrosophila melanogaster? *J. Comp. Physiol. A Neuroethol. Sens. Neural Behav. Physiol.* 1988 Sep;162(5):581–588. 10.1007/BF01342633

[126] Götz KG Hengstenberg B Biesinger R : Optomotor control of wing beat and body posture in drosophila. *Biol. Cybern.* 1979 Nov;35(2):101–112. 10.1007/BF00337435

[127] Alexandrescu A Carew TJ : Postsynaptic effects of cysteine-rich neurotrophic factor in the induction of activity-dependent long-term facilitation in. *Learn. Mem.* 2020 Apr;27(4):124–129. 10.1101/lm.051011.119 32179654 PMC7079570

[128] Hu J Adler K Farah CA : Cell-Specific PKM Isoforms Contribute to the Maintenance of Different Forms of Persistent Long-Term Synaptic Plasticity. *J. Neurosci.* 2017 Mar 8;37(10):2746–2763. 10.1523/JNEUROSCI.2805-16.2017 28179558 PMC5354326

[129] Hu JY Levine A Sung YJ : cJun and CREB2 in the postsynaptic neuron contribute to persistent long-term facilitation at a behaviorally relevant synapse. *J. Neurosci.* 2015 Jan 7;35(1):386–395. 10.1523/JNEUROSCI.3284-14.2015 25568130 PMC4287154

[130] Choi YB Kadakkuzha BM Liu XA : Huntingtin is critical both pre- and postsynaptically for long-term learning-related synaptic plasticity in Aplysia. *PLoS One.* 2014 Jul 23;9(7):e103004. 10.1371/journal.pone.0103004 25054562 PMC4108396

[131] Jin I Kandel ER Hawkins RD : Whereas short-term facilitation is presynaptic, intermediate-term facilitation involves both presynaptic and postsynaptic protein kinases and protein synthesis. *Learn. Mem.* 2011 Feb;18(2):96–102. 10.1101/lm.1949711 21245210 PMC3032577

[132] Reissner KJ Pu L Schaffhausen JH : A novel postsynaptic mechanism for heterosynaptic sharing of short-term plasticity. *J. Neurosci.* 2010 Jun 30;30(26):8797–8806. 10.1523/JNEUROSCI.4767-09.2010 20592201 PMC2911352

[133] Villareal G Li Q Cai D : Role of protein kinase C in the induction and maintenance of serotonin-dependent enhancement of the glutamate response in isolated siphon motor neurons of Aplysia californica. *J. Neurosci.* 2009 Apr 22;29(16):5100–5107. 10.1523/JNEUROSCI.4149-08.2009 19386905 PMC2755541

[134] Glanzman DL : New tricks for an old slug: the critical role of postsynaptic mechanisms in learning and memory in Aplysia. *Prog. Brain Res.* 2008;169:277–292. 10.1016/S0079-6123(07)00017-9 18394481 PMC2855241

[135] Bougie JK Cai D Hastings M : Serotonin-induced cleavage of the atypical protein kinase C Apl III in Aplysia. *J. Neurosci.* 2012 Oct 17;32(42):14630–14640. 10.1523/JNEUROSCI.3026-11.2012 23077049 PMC6621423

[136] Cai D Chen S Glanzman DL : Postsynaptic regulation of long-term facilitation in Aplysia. *Curr. Biol.* 2008 Jun 24;18(12):920–925. 10.1016/j.cub.2008.05.038 18571411 PMC2711037

[137] Fulton D Condro MC Pearce K : The potential role of postsynaptic phospholipase C activity in synaptic facilitation and behavioral sensitization in Aplysia. *J. Neurophysiol.* 2008 Jul;100(1):108–116. 10.1152/jn.90389.2008 18480365 PMC2493496

[138] Wolpaw JR Lee CL : Memory traces in primate spinal cord produced by operant conditioning of H-reflex. *J. Neurophysiol.* 1989 Mar;61(3):563–572. 10.1152/jn.1989.61.3.563 2709100

[139] Wolpaw JR Carp JS Lee CL : Memory traces in spinal cord produced by H-reflex conditioning: effects of post-tetanic potentiation. *Neurosci. Lett.* 1989 Aug 14;103(1):113–119. 10.1016/0304-3940(89)90495-3 2779852

[140] Wang Y Pillai S Wolpaw JR : Motor learning changes GABAergic terminals on spinal motoneurons in normal rats. *Eur. J. Neurosci.* 2006 Jan;23(1):141–150. 10.1111/j.1460-9568.2005.04547.x 16420424

[141] Carp JS Wolpaw JR : Motoneuron plasticity underlying operantly conditioned decrease in primate H-reflex. *J. Neurophysiol.* 1994 Jul;72(1):431–442. 10.1152/jn.1994.72.1.431 7965025

[142] Carp JS Chen XY Sheikh H : Operant conditioning of rat H-reflex affects motoneuron axonal conduction velocity. *Exp. Brain Res.* 2001 Jan;136(2):269–273. 10.1007/s002210000608 11206290

[143] Kaneko N Sasaki A Yokoyama H : Effects of action observation and motor imagery of walking on the corticospinal and spinal motoneuron excitability and motor imagery ability in healthy participants. *PLoS One.* 2022 Apr 18;17(4):e0266000. 10.1371/journal.pone.0266000 35436303 PMC9015126

[144] Simonyan K Avetisyan L Isoyan A : Plasticity in Motoneurons Following Spinal Cord Injury in Fructose-induced Diabetic Rats. *J. Mol. Neurosci.* 2022 Apr;72(4):888–899. 10.1007/s12031-021-01958-9 35083665

[145] Rochat C Bernard-Marissal N Källstig E : Astrocyte-targeting RNA interference against mutated superoxide dismutase 1 induces motoneuron plasticity and protects fast-fatigable motor units in a mouse model of amyotrophic lateral sclerosis. *Glia.* 2022 May;70(5):842–857. 10.1002/glia.24140 34978340 PMC9303637

[146] Wolpaw JR : Harnessing neuroplasticity for clinical applications. *Brain.* 2012 Apr;135(Pt 4):e215. author reply e216. 10.1093/brain/aws017 22374936 PMC3326250

[147] Eftekhar A Norton JJS McDonough CM : Retraining Reflexes: Clinical Translation of Spinal Reflex Operant Conditioning. *Neurotherapeutics.* 2018 Jul;15(3):669–683. 10.1007/s13311-018-0643-2 29987761 PMC6095771

[148] Kane NS Robichon A Dickinson JA : Learning without performance in PKC-deficient Drosophila. *Neuron.* 1997 Feb;18(2):307–314. 10.1016/S0896-6273(00)80270-6 9052800

[149] Drier EA Tello MK Cowan M : Memory enhancement and formation by atypical PKM activity in Drosophila melanogaster. *Nat. Neurosci.* 2002 Apr;5(4):316–324. 10.1038/nn820 11914720

[150] Michel M Green CL Lyons LC : PKA and PKC are required for long-term but not short-term in vivo operant memory in Aplysia. *Learn. Mem.* 2011 Jan;18(1):19–23. 10.1101/lm.2026311 21169419 PMC3023968

[151] Cai D Pearce K Chen S : Protein kinase M maintains long-term sensitization and long-term facilitation in aplysia. *J. Neurosci.* 2011 Apr 27;31(17):6421–6431. 10.1523/JNEUROSCI.4744-10.2011 21525283 PMC3102530

[152] Lyons LC Gardner JS Lentsch CT : Differential role of calpain-dependent protein cleavage in intermediate and long-term operant memory in Aplysia. *Neurobiol. Learn. Mem.* 2017 Jan;137:134–141. 10.1016/j.nlm.2016.11.018 27913293 PMC6179366

[153] Hu J Ferguson L Adler K : Selective Erasure of Distinct Forms of Long-Term Synaptic Plasticity Underlying Different Forms of Memory in the Same Postsynaptic Neuron. *Curr. Biol.* 2017 Jul 10;27(13):1888–99.e4. 10.1016/j.cub.2017.05.081 28648820 PMC5546621

[154] Farley J Schuman E : Protein kinase C inhibitors prevent induction and continued expression of cell memory in Hermissenda type B photoreceptors. *Proc. Natl. Acad. Sci. USA.* 1991 Mar 1;88(5):2016–2020. 10.1073/pnas.88.5.2016 2000409 PMC51157

[155] Etcheberrigaray R Matzel LD Lederhendler II : Classical conditioning and protein kinase C activation regulate the same single potassium channel in Hermissenda crassicornis photoreceptors. *Proc. Natl. Acad. Sci. USA.* 1992 Aug 1;89(15):7184–7188. 10.1073/pnas.89.15.7184 1496012 PMC49670

[156] Muzzio IA Talk AC Matzel LD : Incremental redistribution of protein kinase C underlies the acquisition curve during in vitro associative conditioning in Hermissenda. *Behav. Neurosci.* 1997;111(4):739–753. 10.1037/0735-7044.111.4.739 9267651

[157] Blackwell KT : Subcellular, cellular, and circuit mechanisms underlying classical conditioning in Hermissenda crassicornis. *Anat. Rec. B New Anat.* 2006 Jan;289(1):25–37. 10.1002/ar.b.20090 16437555 PMC2778840

[158] Gao PP Goodman JH Sacktor TC : Persistent Increases of PKMζ in Sensorimotor Cortex Maintain Procedural Long-Term Memory Storage. *iScience.* 2018 Jul 27;5:90–98. 10.1016/j.isci.2018.07.002 30240648 PMC6123865

[159] Soriano EV Ivanova ME Fletcher G : aPKC Inhibition by Par3 CR3 Flanking Regions Controls Substrate Access and Underpins Apical-Junctional Polarization. *Dev. Cell.* 2016 Aug 22;38(4):384–398. 10.1016/j.devcel.2016.07.018 27554858 PMC4998004

[160] Wieschaus E Noell E : Specificity of embryonic lethal mutations in Drosophila analyzed in germ line clones. *Rouxs Arch. Dev. Biol.* 1986 Jan;195(1):63–73. 10.1007/BF00444042 28305278

[161] Zhang L Wei X : The Roles of Par3, Par6, and aPKC Polarity Proteins in Normal Neurodevelopment and in Neurodegenerative and Neuropsychiatric Disorders. *J. Neurosci.* 2022 Jun 15;42(24):4774–4793. 10.1523/JNEUROSCI.0059-22.2022 35705493 PMC9188383

[162] Zha C Sossin WS : The molecular diversity of plasticity mechanisms underlying memory: An evolutionary perspective. *J. Neurochem.* 2022 Dec;163(6):444–460. 10.1111/jnc.15717 36326567

[163] Rocca DL Wilkinson KA Henley JM : SUMOylation of FOXP1 regulates transcriptional repression via CtBP1 to drive dendritic morphogenesis. *Sci. Rep.* 2017 Apr 13;7(1):877. 10.1038/s41598-017-00707-6 28408745 PMC5429823

[164] Bowers JM Perez-Pouchoulen M Roby CR : Androgen modulation of Foxp1 and Foxp2 in the developing rat brain: impact on sex specific vocalization. *Endocrinology.* 2014 Dec;155(12):4881–4894. 10.1210/en.2014-1486 25247470 PMC4239422

[165] Teramitsu I White SA : FoxP2 regulation during undirected singing in adult songbirds. *J. Neurosci.* 2006 Jul 12;26(28):7390–7394. 10.1523/JNEUROSCI.1662-06.2006 16837586 PMC2683919

[166] Thompson CK Schwabe F Schoof A : Young and intense: FoxP2 immunoreactivity in Area X varies with age, song stereotypy, and singing in male zebra finches. *Front. Neural. Circuits.* 2013 Feb 28;7:24. 10.3389/fncir.2013.00024 23450800 PMC3584353

[167] Chen Q Heston JB Burkett ZD : Expression analysis of the speech-related genes FoxP1 and FoxP2 and their relation to singing behavior in two songbird species. *J. Exp. Biol.* 2013 Oct 1;216(Pt 19):3682–3692. 10.1242/jeb.085886 24006346 PMC3763803

[168] Xiao L Merullo DP Koch TMI : Expression of FoxP2 in the basal ganglia regulates vocal motor sequences in the adult songbird. *Nat. Commun.* 2021 May 11;12(1):2617. 10.1038/s41467-021-22918-2 33976169 PMC8113549

[169] Kuo HY Chen SY Huang RC : Speech- and language-linked FOXP2 mutation targets protein motors in striatal neurons. *Brain.* 2023 May 4;146:3542–3557. 10.1093/brain/awad090 37137515 PMC10393416

