# Supplementary material for: Wings of Change: aPKC/FoxP-dependent plasticity in steering motor neurons underlies operant self-learning in Drosophila
Source: F1000Res. 2024 Jun 11;13:116. Originally published 2024 Feb 19. [Version 2] doi: 10.12688/f1000research.146347.2 (PMC11109550; doi:10.12688/f1000research.146347.2)
Supplement: Peer review history

# Reviewer report: Reviewer response for version 1

**DOI:** 10.5256/f1000research.160424.r258493

**Published:** 2024-05-10

**Authors:** Wayne Sossin

**Affiliations:** 1 McGill University, Montreal, QC, Canada

The paper makes important contributions to the field of molecular mechanisms underlying memory formation. I had previously reviewed this paper for a separate journal and this revised version has addressed all the issues I previously had raised and I have no more issues to raise.

Is the work clearly and accurately presented and does it cite the current literature?

Yes

If applicable, is the statistical analysis and its interpretation appropriate?

Yes

Are all the source data underlying the results available to ensure full reproducibility?

Yes

Is the study design appropriate and is the work technically sound?

Yes

Are the conclusions drawn adequately supported by the results?

Yes

Are sufficient details of methods and analysis provided to allow replication by others?

Yes

Reviewer Expertise:

Molecular mechanisms of memory formation and maintenance.

I confirm that I have read this submission and believe that I have an appropriate level of expertise to confirm that it is of an acceptable scientific standard.

# Reviewer report: Reviewer response for version 1

**DOI:** 10.5256/f1000research.160424.r258492

**Published:** 2024-04-17

**Authors:** Kathrin Vogt

**Affiliations:** 1 Universität Konstanz, Konstanz, Germany

Ehweiner et al. describe a potential neural circuit mechanism underlying operant motor learning in *Drosophila melanogaster*. They trained flies to avoid certain flight directions in a flight simulator by punishing steering maneuvers either to the right or left.  In line with previous studies, PKC is required for this kind of self-learning, and the current study suggests that specifically the aPKC gene is required in FoxP-positive motor neurons. The overlapping expression of these two genes is described in wing motor neurons in the VNC. To test for further effects of operant conditioning on flight behavior, the authors test if the optomotor response is affected after training and find a preference shift, suggesting that motor learning does even affect this generally very hardwired and stereotypic behavioral response. Further experimental data investigates the role of FoxP in the brain and the temporal requirements of adult-restricted FoxP knock-out induction.

 The data presented in this manuscript suggests a plastic mechanism, dependent on FoxP and aPKC, within wing motor neurons of the fly required for operant motor learning. The authors provided all raw data and performed rigorous statistical analysis. However, some of the data shows weak phenotypes and thus, excluding a role for some of the genes tested based on a single negative result seems not to be appropriate (PKC53, Baz, Kibra). The authors discuss some of these possibilities, however more experiments are required. Several manipulations in more refined sets of neurons during development might also avoid compensation mechanisms kicking in or the problem of prolonged viability of proteins.

 The effect of motor learning on OMR is very interesting and it will be exciting to see if FoxP is required for the OMR shift, or if it only affects operant learning and the two behaviors are modulated separately.

 Figure 1: aPKC is required in FoxP positive and C380-GAL4 neurons – but the effect upon whole brain KO is small and they still show significant learning. PKC53 KO shows also significant learning is not further tested. To confirm that specifically aPKC is required, the authors should also perform a more restricted manipulation with PKC53 KO -either in FoxP or C380 labeled neurons. Did the authors look at expression patterns of PKC53 in the VNC?

 Figure 2: FoxP expression in the VNC overlaps with aPKC. FoxP is also expressed in many motor neurons. However, as there are many FoxP cells without aPKC expression, the figure cannot clearly indicate that aPKC and FoxP are expressed in the same specific wing motor neurons.

 Figure 3: Co-expression of aPKC and FoxP in specific neuromuscular synapses onto wing muscle cells. It will be interesting to see if the found genes will be specifically required in different wing motor neurons – potentially also dependent on the motor task.

 Figure 4: Flies that have been trained with the operant motor learning paradigm, also have been tested for OMR before and after training. After training, their preference towards the punished side is shifted according to the trained behavior. Maybe the authors could even increase the OMR effect with a more difficult OMR task. Potentially, strong OMR stimuli also induce strong OMR responses, however, if OMR stimuli are less salient or lower in contrast, the flies could maybe more easily suppress specific flight directions. This might enhance the training effect.

 Figure 5: The authors show that FoxP KO in the brain does not influence motor learning. aPKC also seems to have a strong expression in the brain and might be additionally required for plasticity there, however, this has not been tested.

 Figure 6: Here, the authors show that a FoxP-KO restricted to adult flies only shows a phenotype after 14 days. Overall, older flies seem to get worse in operant conditioning. Can the authors exclude that this effect is due to the older age of flies – which might make them more vulnerable to manipulations or weak expression?

 Figure 7: KO of Baz and Kibra in all neurons = no effect on operant learning. In addition to compensation mechanisms, it might be that a cell-specific KO also here might reveal a phenotype – similar to aPKC KO in all neurons, which still showed significant learning. In Figure 6, the authors showed that FoxP adult restricted KO did not reveal any phenotype, could the same problem arise here – maybe the authors can test these effects also in older flies.

 Minor comments:

 Figure 2:

- C2 = The overlap between aPKC and FoxP is very hard to see – a zoom-in with both channels separate would be helpful. Same for Panels D.
- As there are different drivers for GFP across panels, please indicate this in the labels in the figure.
- In Panel D, D42-GAL4 was used to label wing MNs. What is the difference between c380-GAL4 used for behavior and D42-GAL4? Also, D42-GAL4 is not listed in the methods.

Figure 4:

- Colors in the legend are assigned as yellow and green for panel A4/B4, please change this to blue and red.

Is the work clearly and accurately presented and does it cite the current literature?

Yes

If applicable, is the statistical analysis and its interpretation appropriate?

Yes

Are all the source data underlying the results available to ensure full reproducibility?

Yes

Is the study design appropriate and is the work technically sound?

Yes

Are the conclusions drawn adequately supported by the results?

Partly

Are sufficient details of methods and analysis provided to allow replication by others?

Yes

Reviewer Expertise:

Neurogenetics, Neuroethology, Drosophila, Neuromodulation, Learning & Memory

I confirm that I have read this submission and believe that I have an appropriate level of expertise to confirm that it is of an acceptable scientific standard, however I have significant reservations, as outlined above.

# Reviewer report: Reviewer response for version 1

**DOI:** 10.5256/f1000research.160424.r258497

**Published:** 2024-04-04

**Authors:** Efthimios M C Skoulakis

**Affiliations:** 1 Institute for Fundamental Biomedical Research, Biomedical Sciences Research Centre Alexander Fleming, Vari, Greece

The manuscript by Ehweiner et al advances previous findings of the Brembs lab. They provide evidence that the atypical PKC with FoxP positive wing steering neurons is necessary for operant self-learning as the authors define it.

 The manuscript is rather long, and the discussion should be shortened as to not reiterate results.

 Although I have no argument that the phenotypes, they see are consistent with a performance reduction in the trained operant response, I am uncertain about the following:

 1. Can the authors differentiate expression of the learned steering response from the site of actual integration of signals required to change the base line response? In other words, are the motor neurons identified required for expression/performance of the maneuvers necessary to demonstrate the learned behavior?   But is this the actual locus where the learning occurs? If so, what are the afferent neurons encoding the punishment that should impinge on these motoneurons? 

 Is it possible that the site of learning is elsewhere within the nervous system a possibility also consistent with the Elav-driven knock out data? This should at least be discussed in the appropriate section.

 Perhaps not at this point but differential (side specific) photo activation of the identified neurons might provide necessary evidence on their sufficiency to drive optomotor bias.

 2. I do not understand the result in Fig 6. How can the effect be 12 days after removal of the RU486 inducer?!!! If I remember correctly from the original paper detailing the GS system, the effect of RU486 wears out within 24 hrs post removal of the drug. However, does induction poison the FoxP neurons and it takes that long for them to degenerate? Are the neurons there post treatment? 

 Have the authors tried RU486 induction for more than 2 days??

 Minor points:

 1. Please list on table 1 where each driver is expressed.

 2. On page 8 in the opening paragraph of the results section. Please summarize (for the naive reader) previous findings that led you to look specifically at the FoxP neurons.

Is the work clearly and accurately presented and does it cite the current literature?

Yes

If applicable, is the statistical analysis and its interpretation appropriate?

Yes

Are all the source data underlying the results available to ensure full reproducibility?

Yes

Is the study design appropriate and is the work technically sound?

Yes

Are the conclusions drawn adequately supported by the results?

Partly

Are sufficient details of methods and analysis provided to allow replication by others?

Yes

Reviewer Expertise:

Associative and non associative learning and memory and associated disorders modeled in Drosophila

I confirm that I have read this submission and believe that I have an appropriate level of expertise to confirm that it is of an acceptable scientific standard, however I have significant reservations, as outlined above.
